# Gas exclusion zones in Type II porous liquids

**DOI:** 10.1039/d5sc06588g

**Published:** 2025-11-19

**Authors:** Cathal F. Kelly, Sergio F. Fonrouge, José L. Borioni, Mario G. Del Pópolo, Émer M. F. Rooney, Deborah E. Crawford, K. Travis Holman, Stuart L. James

**Affiliations:** a Department of Chemistry and Chemical Engineering, Queen's University Belfas David Keir Building, Stranmillis Road Belfast UK s.james@qub.ac.uk; b ICB-CONICET, Facultad de Ciencias Exactas y Naturales, Universidad Nacional de Cuyo Padre Jorge Contreras 1300 Mendoza Argentina; c Instituto de Investigaciones en Físico-Química de Cordoba (INFIQC-CONICET), Departamento de Química Orgánica, Facultad de Cienias Químicas, Universidad Nacional de Córdoba Argentina; d School of Chemistry, The University of Birmingham Edgbaston Birmingham BT15 2TT UK; e Department of Chemistry, Georgetown University Box 571227 Washington DC 20057 USA

## Abstract

Porous liquids combine permanent porosity with fluidity and may ultimately find uses which are not possible for conventional liquids or porous solids. An important general characteristic of porous liquids studied to date is that they exhibit very high gas solubilities. Here, we examine this aspect in more detail than has been done previously, in particular with regard to CO_2_ and CH_4_ solubility in the Type II porous liquid Noria_OEt_@15C5 (15C5 = 15-crown-5). Whilst this porous liquid exhibits increased CH_4_ solubility compared to neat 15-crown-5, counterintuitively it actually exhibits equal or lower CO_2_ solubility than the neat solvent 15C5 at pressures above 1 bar. Molecular dynamics modelling reveals that although the pore space does provide a good binding site for gas molecules, there is an ‘exclusion zone’ around the pore space within which binding of CO_2_ molecules is disfavoured compared to binding within the bulk solvent. The unfavourable binding in this region arises from a number of effects, including (i) steric exclusion from the bulky covalent framework of the Noria_OEt_ host, and (ii) ordering of 15C5 solvent molecules in the solvation shell around the Noria_OEt_. The first porous liquid to be based on the host Cryptophane-A, Cryptophane-A@Cyrene, was prepared in the expectation that the smaller framework bulk of Cryptophane-A compared to that of Noria_OEt_ should result in a smaller exclusion zone. Correspondingly, this porous liquid did indeed exhibit improved CO_2_ uptake compared to its neat solvent, supporting the assertion that the exclusion zone is at least in part due to exclusion of gas from the framework of the host. Overall, the work provides a more sophisticated understanding of gas solubility in Type II PLs and suggests some additional design considerations for achieving high solubility for a given gas. It also shows that, as well as being able to increase the solubility of certain gases PLs can also conceivably be designed to suppress the solubility of gases under some conditions, which could be useful in tuning selective dissolution.

## Abstract 2

Although gas binding in the pores of porous liquids (PLs) is favourable, we show for the first time that there is an exclusion zone around the pore which decreases gas uptake. We introduce the first Cryptophane-based PL to reduce this effect.

## Graphical abstract

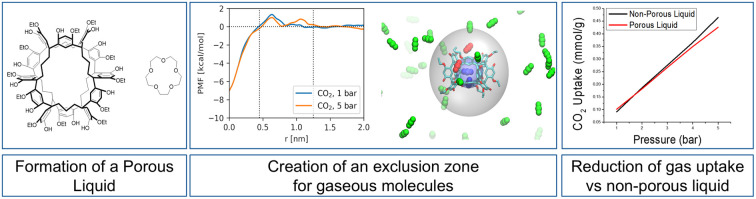

## Introduction

Porous liquids (PLs) are a new class of materials which are capable of efficient and continuous separation of gases due to their combination of fluidity and permanent porosity. The presence of pores increases gas solubility through physisorption and can potentially do this on a size- and shape-selective basis.^1–4^

Type II porous liquids (T2PLs) consist of rigid, empty host species dissolved in size-excluded solvents. Rigidity of the host is important, as flexible hosts could collapse upon the removal of guest molecules which could negate the porosity. The hosts should also be highly soluble to maximise the porosity of the porous liquid. Several hosts have been identified as suitable generating T2PLs including imino-spherand organic cages, Noria_OEt_ and metal–organic cages (MOCs).^5,6^

Studies of Type II PLs to date have all found that the solubility of gases in these phases is greatly increased with respect to the neat solvent, which is expected from the presence of permanent pores. In particular, from Scaled Particle Theory, the main energy penalty to dissolution of a solute is the energy required to form a notional ‘cavity’ (*i.e.* pore) in the solvent in which the solute can be accommodated.^7^ In PLs, the pore is pre-formed by the molecular host and so this energy penalty to form a pore is negated.

**Fig. 1:**
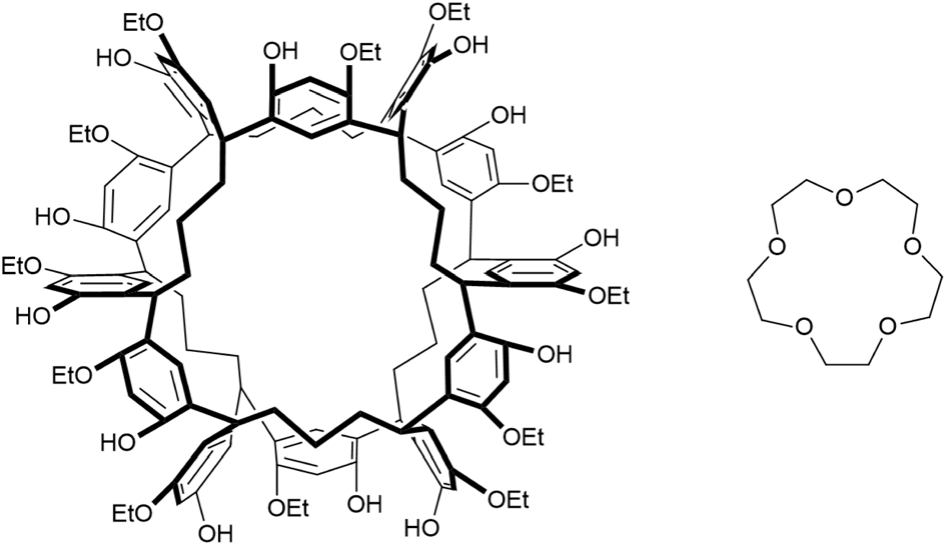
Structures of Noria_OEt_ (left) and the size-excluded solvent 15-crown-5 (15C5, right) which were used to form the Type II porous liquid Noria_OEt_@15C5.

**Fig. 2:**
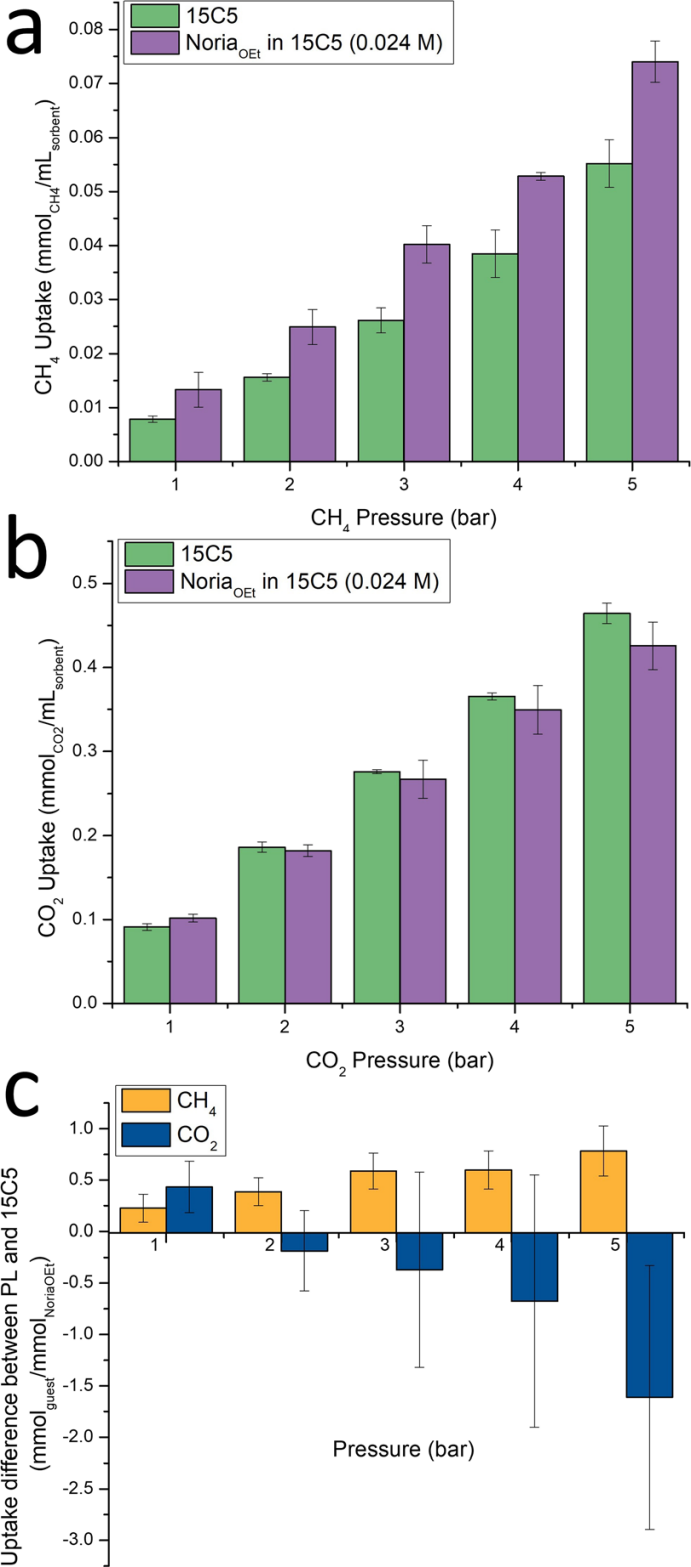
Gas solubilities in Noria_OEt_@15C5 and pure 15C5 from 1-5 bar. (a) CH_4_, (b) CO_2_, (c) the ‘apparent occupancy’ of the Noria_OEt_ host, specifically the difference between the gas solubilities in 15C5 and Noria_OEt_@15C5, expressed as a mole ratio.

Noria_OEt_ (Fig. 1, (left)) was shown by Alexander *et al.* to be an effective host for Type II PLs due to its rigidity, internal cavity, ease of synthesis and chemical robustness.^8^ It has good solubility in 15-crown-5 (15C5) (Fig. 1, (right)) which is often used in Type II PLs as a size-excluded solvent. The resulting solution, Noria_OEt_@15C5-24 mM (hereafter simply Noria_OEt_@15C5) was concluded to be a porous liquid because of the increased solubility of CH_4_ compared to that in pure 15C5, and the presence of pores was supported by detailed modelling using molecular dynamics. The CH_4_ solubility data are reproduced in Fig. 2a. The PL consistently exhibits greater CH_4_ solubility than does pure 15C5 at pressures from 1–5 bar and the difference in solubility increases with pressure.

In this paper, we have explored the solubility of CO_2_ in Noria_OEt_@15C5 and made some counter-intuitive findings that increase our understanding of gas uptake in Type II PLs. We also report a new T2PL, Cryptophane-A@Cyrene.

## Results and discussion

### Porous liquid synthesis and gas uptake measurements

Noria_OEt_ and Noria_OEt_@15C5 (24 mM) were prepared as described previously.^8^ Briefly, Noria_OEt_ was dissolved in 15C5 by gentle heating. All characterisation data for Noria_OEt_ and Noria_OEt_@15C5 were in accord with the previous work.^8^ The resulting solution was then left overnight in a refrigerator to allow for any potential precipitation and ensure that the solution was not super-saturated. CO_2_ solubility in Noria_OEt_@15C5 was measured at room temperature from 1–5 bar using the same isobaric method used previously for Noria_OEt_@15C5 and for other porous liquids as described elsewhere and in the SI.^8–10^ For comparison, the analogous CO_2_ solubility data for pure 15C5 were also measured and are shown in Fig. 2b. All data were measured in triplicate and error bars indicate standard deviations.

At 1 bar, Noria_OEt_@15C5 shows greater CO_2_ solubility than does 15C5, as expected for a porous liquid. However, unexpectedly, from 2–5 bar the PL exhibits equal or lower CO_2_ solubility than 15C5. Given the standard deviations in the data care must be taken in comparing solubilities in 15C5 and Noria_OEt_@15C5 at specific pressures. However, the data suggest a trend of Noria_OEt_@15C5 becoming a progressively worse CO_2_ solvent than 15C5 as pressure increases.

The contrast in behaviour between CH_4_ and CO_2_ is emphasized in Fig. 2c, which shows the ‘apparent occupancy’ of the Noria host. Specifically, for each gas, we plot the difference between its solubility in Noria_OEt_@15C5 and in pure 15C5. The data are expressed as the mole ratio of this difference in gas solubility to the amount of Noria_OEt_ present in the PL. Here, the error bars again make it impossible to draw firm conclusions in comparing data between specific pressures within either series. However, this representation emphasises the overall trend that for CH_4_ as pressure increases the PL is increasingly a better solvent than pure 15C5. It also shows that, notably, even at 5 bar the average degree of host occupation is still only *ca.* 0.78 molecules of CH_4_. For CO_2_, at 1 bar the PL is a better solvent than pure 15C5, with an apparent occupancy of 0.43, greater than that observed for CH_4_ (0.23), which would be expected from the expected stronger binding of CO_2_ However, above 1 bar pressure Fig. 2c emphasises that the PL becomes increasingly worse than 15C5 as a solvent, as mentioned above. This is highlighted by the fact that the apparent occupancy becomes negative at pressures greater than 1 bar. Therefore, it would seem that for CO_2_ the beneficial effect of the pore only dominates the gas uptake at relatively low pressures (1 bar), and at higher pressures other negative effects appear to dominate.

With regard to these other effects, we initially reasoned that the difference in behaviour between the two gases might reflect the fact that the pure solvent 15C5 is a much better solvent for CO_2_, than for CH_4_ (based on our solubility data the Henry constants, *H*_s_^cp^, are 93.9 × 10^−3^ mol per L per atm and 11.9 × 10^−3^ mol per L per atm for CO_2_ and CH_4_ respectively). In particular, at higher pressures, the dominant effect of the bulky Noria_OEt_ host could be that it effectively replaces a significant number of solvent molecules with a region of space from which the gas molecules are sterically excluded by the bulk of the host's structure. These solvent molecules are much better at dissolving CO_2_ than CH_4_, and thus the observed effect is that the overall CO_2_ solubility is negatively impacted at higher pressures. Effectively, a gas “exclusion zone” exists around the pore due to the host's framework. We also reasoned that this exclusion could potentially extend further, into the solvation shell of Noria_OEt_@15C5, since in this region CO_2_ molecules most compete with Noria_OEt_ for solvation sites (*i.e.* the oxygen centres) of the 15C5 solvent.

### Molecular dynamics simulations

These hypotheses were explored using molecular dynamics (MD) modelling. Simulations were used to examine the spatial distribution of gas molecules in the porous liquid, identifying thermodynamically favoured or disfavoured regions and characterising solvent structuring around the host. Simulation parameters, including force-field details, system preparation, and validation procedures, are provided in the SI.

To explore the free energy landscape for gas positioning around the host, Umbrella Sampling (US) simulations were used to compute the potential of mean force (PMF) for transferring a CO_2_ or CH_4_ molecule from bulk solvent to a radial distance, *r,* from the centre of a Noria_OEt_ cage. This allowed the identification of exclusion zones where gas accumulation is thermodynamically disfavoured (PMF > 0). Unbiased MD simulations were also conducted on a bulk system with the experimental Noria_OEt_-to-solvent mole ratio to capture the spontaneous distribution of gas molecules and identify any depletion zones. Gas concentrations were set according to the experimental solubility values. Additionally, a third set of simulations, performed in the absence of gas, examined solvent structuring around Noria_OEt_ and the distribution of interstitial voids within and around the host. All simulations were carried out at 300 K under 1 bar or 5 bar pressures to assess the impact of hydrostatic pressure on gas exclusion.

**Fig. 3:**
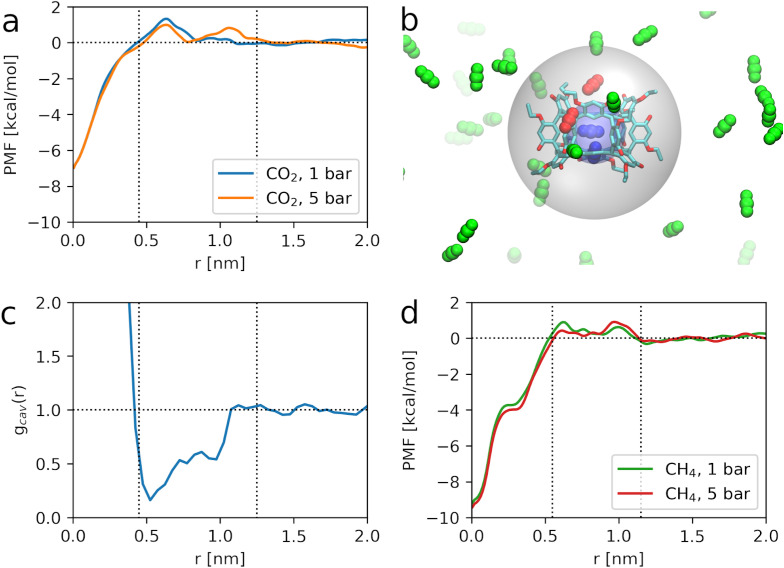
(a and d) Free energy profiles, or PMF, for transferring a CO_2_ or CH_4_ molecule from bulk solvent to a radial distance *r* from a Noria_OEt_ dissolved in 15C5. Three domains, illustrated with dotted lines, are observable from the energy differences relative to the bulk. (b) Snapshot of a simulation of the porous liquid loaded with CO_2_ at 5 bar. The translucent spheres illustrate the inner and outer shells defined from the first two domains in the free energy profiles of CO_2_. CO_2_ molecules inside the inner shell and in the outer shell are depicted in blue and red respectively. (c) Radial distribution function of spherical cavities of radii larger than 0.1 nm relative to the centre of a single Noria_OEt_ cage in 15C5 at 1 bar. Depletion of cavities occurs in a range that is mostly contained in the second domain of the CO_2_ free energy profile, deemed as an exclusion zone.

Simulations at 1 bar reveal that gas molecules do indeed experience exclusion effects in the porous liquid, consistent with the observed experimental reduction in CO_2_ solubility described above. The PMF profiles for CO_2_, shown in Fig. 3a, indicate three distinct regions around Noria_OEt_. As expected, within the host cavity (0 ≤ *r* < 0.45 nm), CO_2_ is strongly stabilised, with an inclusion energy of −7 kcal mol^−1^, explaining why the cavity remains occupied at all pressures considered (see SI, Fig. S14). Beyond *r* > 1.25 nm, the CO_2_ molecules behave as in bulk solvent, where the host molecule has no influence. However, in the intermediate region (0.45 ≤ *r* ≤ 1.25 nm), a wide repulsive barrier of ∼1 kcal mol^−1^ is observed, forming an exclusion zone where the positioning of gas molecules is thermodynamically disfavoured. This repulsion is further enhanced at higher pressures (5 bar).

Radial distribution functions (RDFs) calculated from unbiased MD (see SI, Fig. S15) confirm these findings: the system exhibits a fully occupied host cavity, a gas-depleted exclusion zone (*g*(*r*) < 1), and a solvent-dominated bulk region. This spatial organisation is illustrated in Fig. 3b, where CO_2_ molecules preferentially occupy the host cavity (blue) or disperse in the bulk (green), while the exclusion zone (red) remains sparsely populated. In terms of the geometry of the exclusion zone for CO_2_ and its correspondence to the host shape and size, a radius of 0.45 nm captures the entirety of the host cavity and part of its opening. The outer shell, with a radius of 1.25 nm, encloses the entirety of the Noria_OEt_ host, reaching beyond the methyl endings of its ethoxy groups.

The structural basis of the exclusion zone was further examined by analysing the distribution of voids in and around the host, including the interstitial voids in the space occupied by the solvent and the host cavity itself. To quantify this, we identified regions within the simulation cell where a hard sphere of radius *R* > 0.1 nm could be inserted without overlapping with any atoms (see SI, Section S9 for details). The radial distribution function of these cavities, *g*_cav_(*r*), was then computed relative to the geometric centre of Noria_OEt_.^8^ The results reveal a clear depletion of voids within the same spatial range identified as the exclusion zone for CO_2_, indicating that the packing of solvent molecules around Noria_OEt_ reduces the availability of accessible free volume, thereby restricting gas insertion. This is evident in Fig. 3c, where *g*_cav_(*r*) shows a sharp peak at *r* ≈ 0 nm, corresponding to the intrinsic cavity of the host, while the surrounding region exhibits a marked reduction in void density (*g*_cav_(*r*) < 1) from 0.41 to 1.07 nm, aligning with the spatial extent of the exclusion zone. Incidentally, the maximum in the Noria_OE_-solvent RDF occurs at *r* ≈ 1 nm (SI Fig. S6), where the solvent density is greatest, reinforcing the correlation between solvent structuring and the lack of free space revealed by the void distribution. As might be expected, the largest depletion occurs at 0.5 nm (*g*_cav_(*r*) ≈ 0.2), roughly the distance to the walls. *g*_cav_(*r*) then ramps up to ≈0.5 at 1 nm. The upper limit of the depletion range has a closer match to the beginning of the asymptotic behaviour of the PMF for CO_2_ at 1 bar (Fig. 3a), but that point is also a maximum in the PMF for CO_2_ at 5 bar. Thus, exclusion is not explained entirely by the steric hindrance from Noria_OEt_, and the interplay and competition between solvent molecules and gas molecules in the proximity to the cage must be considered as well. Effectively, within the Noria_OEt_ solvation shell, the 15C5 molecules are more densely packed than in the bulk solvent, restricting the ability to solvate CO_2_ molecules in this region.

Finally, Fig. 3d shows the PMF for a single CH_4_ molecule at 1 bar and 5 bar. CH_4_ experiences a stronger stabilisation in the host cavity, with insertion free energies ranging from −9.2 to −9.4 kcal mol^−1^. More importantly, the exclusion zone is both narrower (0.55 ≤ *r* ≤ 1.15 nm) and less repulsive than that of CO_2_, suggesting that CH_4_ experiences weaker exclusion effects. While the qualitative features of the PMF are similar for both gases, the weaker exclusion for methane is consistent with its greater increase in experimental solubility in the porous liquid system relative to 15C5. It can also be noted that since the Henry constant for CH_4_ in 15C5 is *ca.* 10 times greater than that of CO_2_ (see above), *i.e.* 15C5 is a much poorer solvent for CH_4_ than for CO_2_, the replacement of 15C5 solvent molecules by the bulk of the Noria_OEt_ structure has less of an effect on the overall CH_4_ solubility than it does on the CO_2_ solubility.

### Cryptophane-based porous liquid

**Fig. 4:**
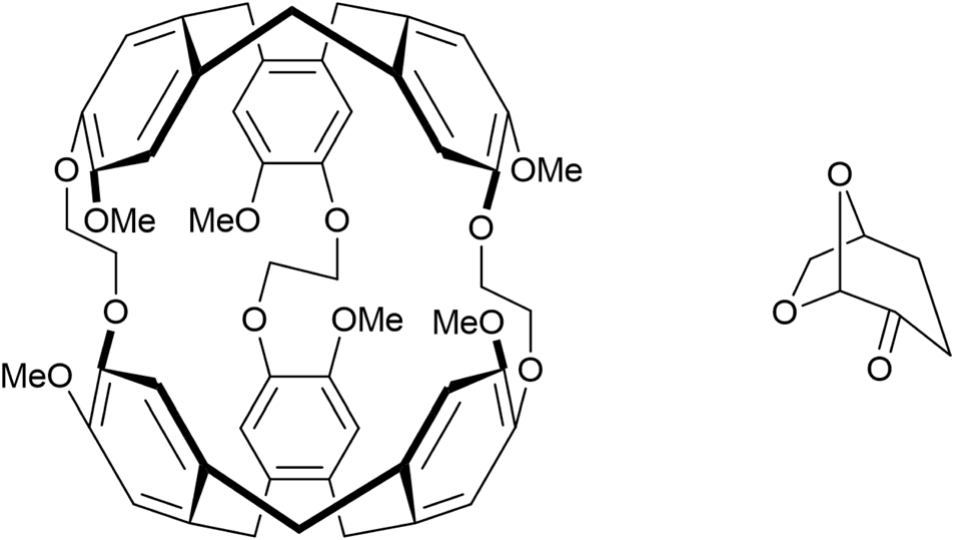
Structures of Cryptophane-A (left) and Cyrene (right).

It follows from the above that the gas uptake of a PL can be maximised by minimising the volume of the exclusion zone. In order to test this hypothesis, Cryptophane-A was explored as a host for a T2PL (Fig. 4, left). Cryptophanes are a class of hosts based on two cyclotribenzylene (CTB) units connected by various linkers. They are potentially appropriate hosts for PLs. Collado *et al.* have computationally simulated Cryptophane-111 dissolved in CH_2_Cl_2_ as a potential PL for encapsulation of CO_2_ and H_2_, and for SO_2_ separation and storage.^11,12^

**Fig. 5:**
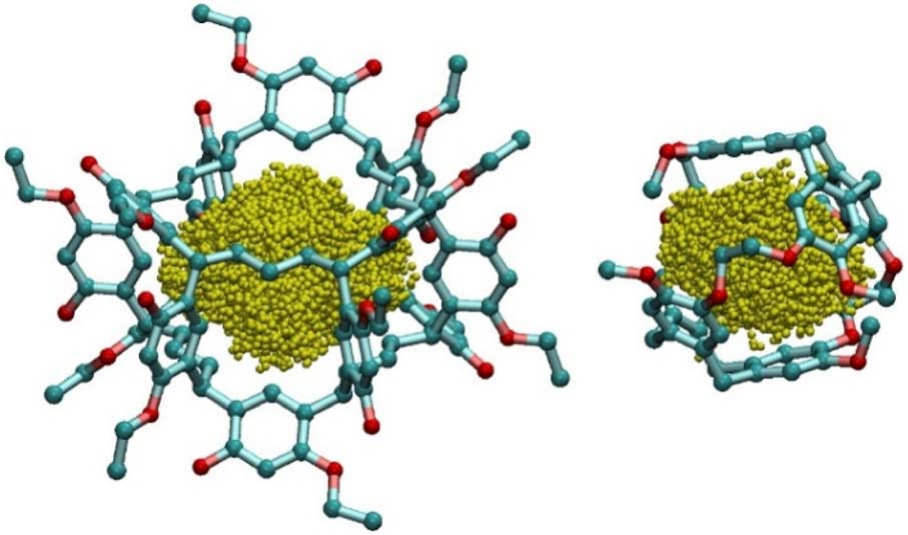
A graphical illustration of cavity volumes of Noria_OEt_ (left) and Cryptophane-A (right). Cavity volumes are illustrated by yellow spheres, each of which represents a sampling point in the simulation. The cavity volumes are similar whilst the total volume of Noria_OEt_ is greater than that of Cryptophane-A.

Using a computational method described in the SI (S13), the van der Waals (vdW) and cavity volumes of Noria_OEt_ and Cryptophane-A were estimated and compared. The structures used were the Noria_OEt_ model that had been generated computationally in the above work and the crystal structure of the Cryptophane-A Xe inclusion complex published by Taratula *et al.*,^13^ with the Xe guest deleted. A Monte Carlo integration scheme was used to calculate the vdW volume of each molecule. For Noria_OEt_, the surface substituents were included in the calculation. Random points were uniformly sampled within a bounding box that enclosed the entire molecule, extended by 4 Å in each spatial direction to avoid truncating peripheral volume. For each point, we checked whether it fell within the van der Waals radius of any atom in the molecule. The vdW volume was then calculated as the fraction of points falling within any vdW sphere, multiplied by the volume of the bounding box. To compute the cavity volume, we first identified the atoms which define the inner cage of the host molecule and constructed the convex hull defined by their coordinates. We then used the same random point set to identify those points that were located within the convex hull but outside the vdW spheres of all atoms. The volume of the cavity was then estimated as the fraction of such points multiplied by the bounding box volume. This approach yields the internal volume that is geometrically enclosed by the cage but not occupied by any atom. These cavity volumes are illustrated in Fig. 5. Noria_OEt_ was found to have a vdW volume of 1793 Å^3^ with a cavity volume of 141 Å^3^. For Cryptophane-A, the vdW volume was 765 Å^3^, and the cavity volume was 124 Å^3^. This cavity volume is comparable to reported values, as discussed by El-Ayle and Holman.^14^ The ratio of cavity volume to vdW volume (here defined as *α*), for each host was calculated to be 7.8% and 15.8% for Noria_OEt_ and Cryptophane-A respectively. The greater *α* value for Cryptophane-A in comparison with Noria_OEt_, combined with the similar cavity volumes of the two hosts, suggests that Cryptophane-A should have a smaller exclusion zone when used to form a PL. This in turn should lead to greater gas solubility than for Noria_OEt_@15C5.

Cryptophane-A was synthesised by the method described by Della-Negra *et al.*^15^ Prior to testing the host solubility in candidate solvents, Cryptophane-A samples were dried under vacuum at 80 °C. Unfortunately, Cryptophane-A was found to be insoluble in 15C5. Therefore, we investigated dihydrolevoglucosanone (Cyrene, Fig. 4, right) as an alternativesolvent. Cyrene is a biorenewable solvent with similar solubility parameters to polar aprotic solvents such as DMF and NMP, suggesting that it is capable of strong solvation. It also has a bulky, globular shape due to its bicyclic structure, suggesting that it should be excluded from pore windows of up to 7 Å. Also, Cyrene has no known toxicity issues and is produced from biomass. Compared to existing solvents for T2PLs, Cyrene is therefore attractive in terms of sustainability and safety. Due to its level of oxygenation, Cyrene was expected to have a similar gas uptake profile to 15C5. Cryptophane-A exhibited good solubility in Cyrene, which actually allowed for the preparation of more concentrated PLs than for Noria in 15C5. This would allow for a clearer evaluation of the effect of a potentially smaller exclusion zone. The resulting 48 mM solution is hereafter referred to as Cryptophane-A@Cyrene.

Since the solubility of gases in pure Cyrene has not previously been reported we first established this, using the same method as described above for Noria_OEt_@15C5.^8^ CH_4_ solubility in Cyrene was found to be 0.006 mmol_CH_4__ per mL at 1 bar, increasing linearly (within error) up to 0.052 mmol_CH_4__ per mL at 5 bar. CO_2_ solubility was measured to be 0.12 mmol_CO_2__ per mL at 1 bar, increasing linearly (within error) to 0.52 mmol_CO_2__ per mL at 5 bar. The good CO_2_/CH_4_ selectivity (17.8 at 1 bar; 10.0 at 5 bar) is as expected for an oxygenated solvent due the favourable interactions between Lewis acidic CO_2_ and the basic oxygen centres, as in commercial polyethylene glycol-diether solvents such as Genosorb for example.^9^ Using the above data, the Henry constants, *H*_s_^cp^, for CH_4_ and CO_2_ in Cyrene were calculated to be 11.8 × 10^−3^ mol per L per atm and 10.3 × 10^−2^ mol per L per atm respectively. These values are similar to those for 15C5 (see above).

**Fig. 6:**
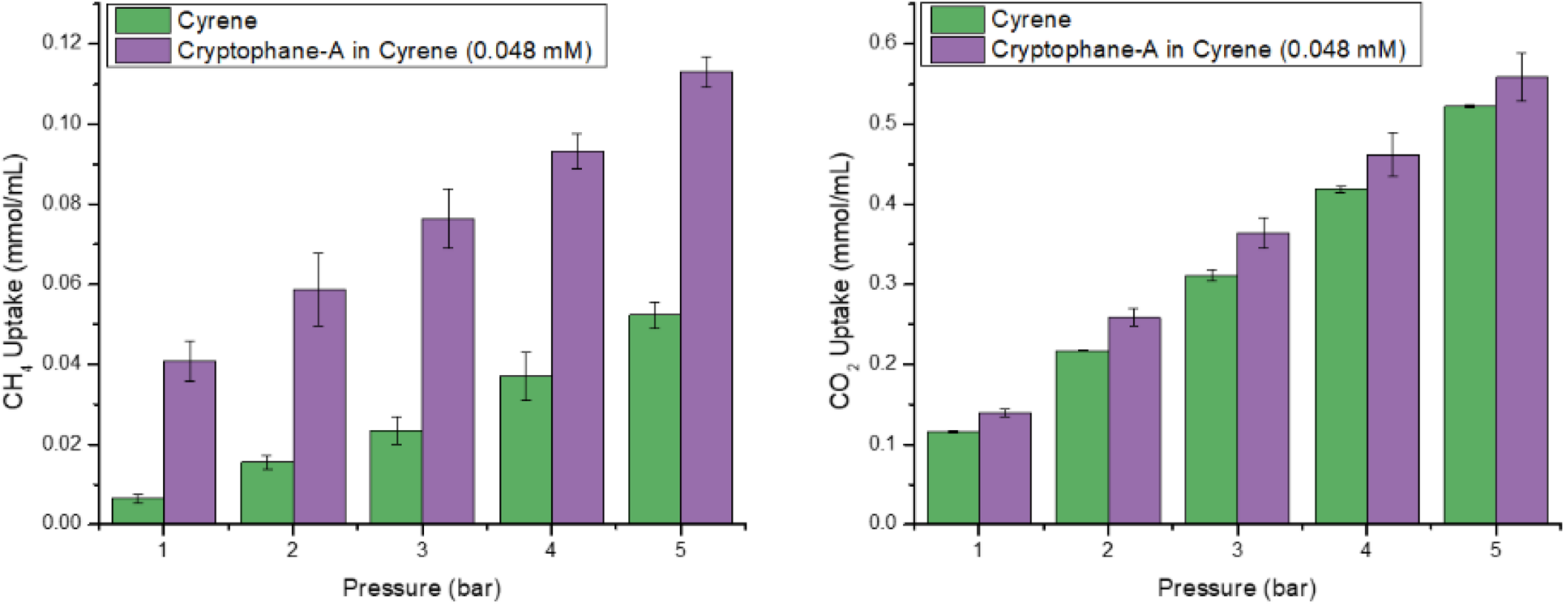
(Left) CH_4_ uptake of Cyrene and of Cryptophane-A in Cyrene from 1-5 bar at 298.15 K. (Right) CO_2_ uptake of Cyrene and of Cryptophane-A in Cyrene from 1–5 bar at 298.15 K.

As expected, CH_4_ was more soluble in the porous liquid Cryptophane-A@Cyrene (*e.g.* 0.04 mmol_CH4_ per mL_PL_ at 1 bar and 0.11 mmol_CH_4__ per mL_PL_ at 5 bar, Fig. 6, left) than in pure Cyrene. The enhancement corresponds to *ca.* 0.7 molecules of CH_4_ per molecule of Cryptophane-A at 1 bar, rising to 1.3 molecules at 5 bar. These average levels of occupancy are consistent with ^1^H-NMR studies conducted by Garel *et al.*^16^ on solid Cryptophane-A, which showed that one molecule of CH_4_ can readily be accommodated within the Cryptophane-A cavity, and potentially two at higher pressures.

**Fig. 7:**
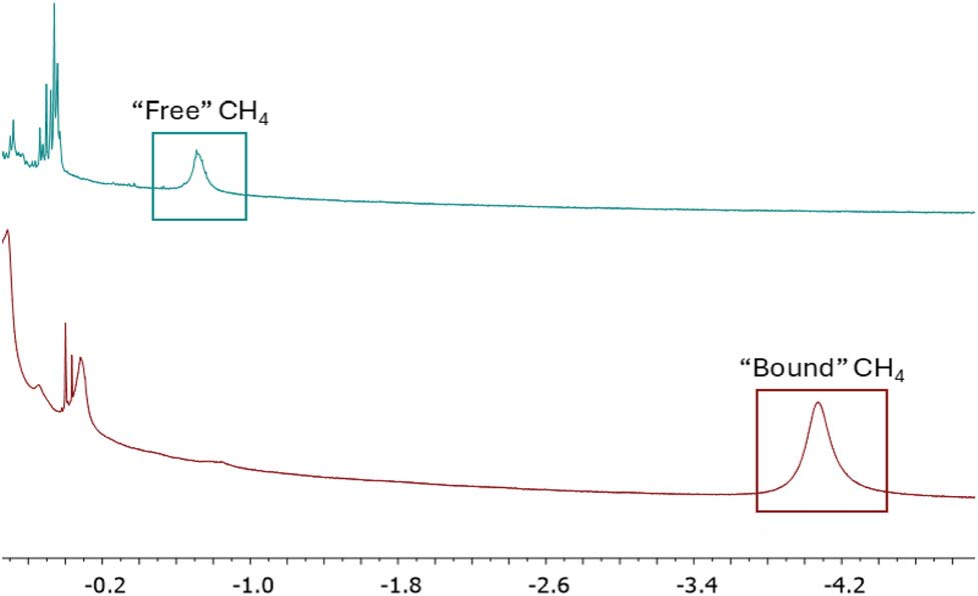
^1^H-NMR spectra of Cyrene (top) and Cryptophane-A@Cyrene (bottom) after having CH_4_ bubbled through the sample. The upfield shift of the CH_4_ signal is characteristic of inclusion within a Cryptophane.

^1^H-NMR spectroscopy confirmed that inclusion of CH_4_ occurs within the cavities of the Cryptophane in the PL. CH_4_ was bubbled through Cyrene in an NMR tube containing a *d*^6^-acetone capillary. A ^1^H-NMR spectrum showed the CH_4_ peak at −0.73 ppm (Fig. 7). A similar experiment conducted using Cryptophane-A@Cyrene in place of neat Cyrene showed a CH_4_ peak at −3.95 ppm. This clear upfield shift is consistent with that reported for the inclusion of CH_4_ in Cryptophane-A by Collet *et al.*^17^ A similar experiment with C_2_H_6_ showed analogous behaviour (Fig. S10 and S11) with inclusion in the Cryptophane host causing an upfield shift from −0.02 ppm to −3.66 ppm.

The CO_2_ solubility in Cryptophane-A@Cyrene was then measured to test the hypothesis that a smaller exclusion zone would increase CO_2_ solubility relative to neat Cyrene. Cryptophane-A@Cyrene did indeed exhibit an enhanced CO_2_ solubility, ranging from 0.14 mmol_CO_2__ per g_PL_ at 1 bar to 0.55 mmol_CO_2__ per g_PL_ at 5 bar. This increase in solubility compared to neat Cyrene corresponds to an apparent occupancy 0.5 at 1 bar. Interestingly, the apparent occupancy reaches a maximum of 1.1 at 3 bar before falling to 0.75 at 5 bar (as discussed further below). Overall, this range of occupancies is intuitively reasonable and similar to that seen for CH_4_.

**Fig. 8:**
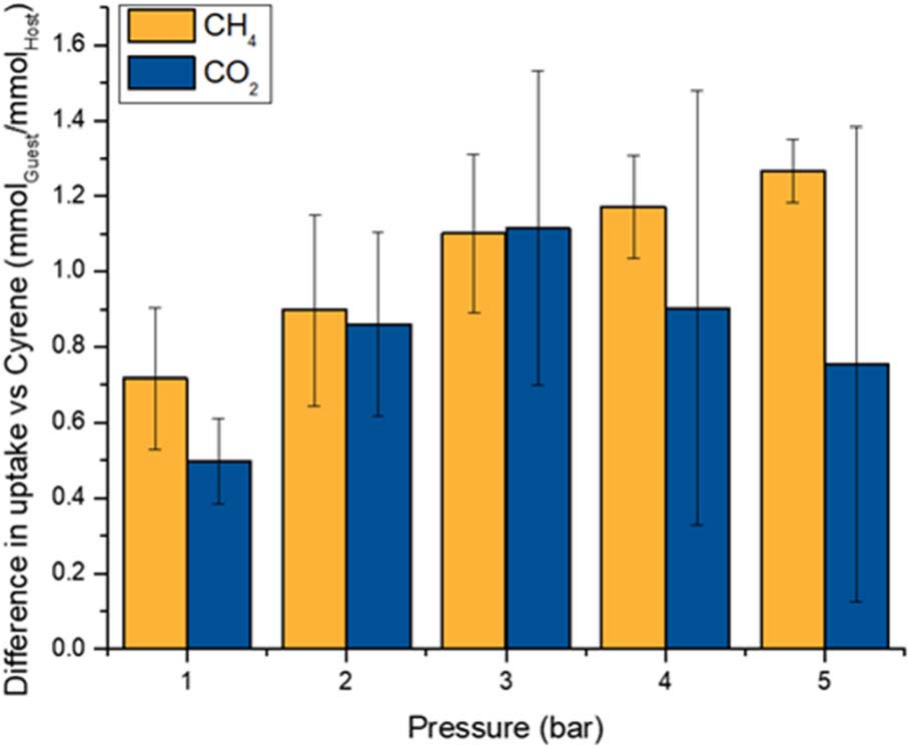
Difference between the gas uptake of Cryptophane-A@Cyrene and Cyrene in terms of mmol_Guest_ per mmol_Host_.

Finally, by analogy with Fig. 2c, it is interesting to plot for each gas the apparent occupancy of the Cryptophane-A hosts in the PL, specifically the difference between the gas's solubility in Cryptophane-A@Cyrene *vs.* pure Cyrene, expressed as a mole ratio of gas to Cryptophane-A in the PL (Fig. 8). As with Noria_OEt_@15C5 (Fig. 2c), the error bars which represent ESDs preclude comparisons between specific data points within each series. However, with regard to overall trends, it is notable, firstly, that the behaviour of CH_4_ is similarly to that in Noria_OEt_@15C5, *i.e.* as pressure increases the PL becomes progressively a better solvent than Cyrene, with steadily increasing apparent occupancy of the host. However, the behaviour of CO_2_ in this regard is different from that in Noria_OEt_@15C5. Specifically, at lower pressures Cryptophane-A@Cyrene becomes increasingly better as a solvent than Cyrene with increasing pressure. This trend is the opposite to that seen for Noria_OEt_@15C5 (Fig. 2c), and is consistent with the exclusion zone for Cryptophane-A being smaller than for Noria_OEt_. As mentioned above, the apparent occupancy for CO_2_ increases with pressure up to a maximum at 3 bar, above which the trend reverses, reverting to that seen in Fig. 2c (*i.e.* the as pressure increases above 3 bar the apparent occupancy decreases as the PL performs comparatively less well compared to neat Cyrene). This latter behaviour reveals that although the exclusion zone is smaller for Cryptophane-A than for Noria_OEt_, it is still present, although only observed at greater CO_2_ pressures. This is intuitively reasonable since increasing the CO_2_ pressure increases the amount of CO_2_ that would be dissolved in pure Cyrene, and therefore the effect of replacing Cyrene solvent molecules by the framework bulk of the Cryptophane (from which CO_2_ molecules are sterically excluded) becomes more apparent at high pressure.

Overall, a key aspect is that the enhanced solubility of CO_2_ in Cryptophane-A@Cyrene compared to that in neat Cyrene supports the hypothesis that by reducing the size of the exclusion zone in a porous liquid, the gas solubility in the porous liquid can be increased relative to the neat solvent.

## Conclusions

In summary, experimental gas solubility measurements show that, counterintuitively, CO_2_ solubility in Noria_OEt_@15C5 is equal or even reduced compared to that in its neat solvent, 15C5, despite the presence of empty pores. To our knowledge such behaviour has not previously been observed for a porous liquid. Simulations suggest that although the Noria_OEt_ pore provides a strongly favourable site for enhanced gas binding, there exists a gas exclusion zone surrounding the pore, in which binding is disfavoured. The exclusion zone arises from two aspects, (i) steric exclusion of gas molecules from the region defined by the bulky host framework (*i.e.* replacement of solvent molecules by the bulk of the host) and (ii) solvent ordering in the solvation shell of the host. This latter aspect consists of a complex interplay between the distribution of interstitial voids around the host and intermolecular interactions involving the gas, solvent, and host. In the MD simulations conducted these latter interactions are inherently tied to the force-field model used. Although the force-field parameters yield gas insertion energies in the host cavity comparable to DFT calculations (see Section S5 of the SI), it can be borne in mind that small discrepancies in host–solvent–gas interactions may alter the delicate balance of forces governing gas exclusion. Given that the energy barriers associated with exclusion are relatively small, future implementations of the force-field may require fine-tuning to fully capture subtle solvation effects that distinguish CO_2_ from CH_4_ quantitatively. Nevertheless, within the accuracy of the current model, the simulations are qualitatively consistent with the experimental trends, correctly capturing the stronger exclusion effect for CO_2_ and the weaker, less pronounced effect for CH_4_.

It was also shown experimentally, that by using a porous host which is calculated to have a smaller exclusion zone than Noria_OEt_ (Cryptophane-A), the effect of the exclusion zone can be reduced. Specifically, this was demonstrated by Cryptophane-A@Cyrene, having increased CO_2_ solubility compared to its neat solvent. This latter material is also noteworthy in being the first reported porous liquid based on a Cryptophane host.

In related work, it is interesting to note that non-additive CO_2_ uptake has recently been reported in which iminospherand-based Type 2 PLs demonstrated enhanced CO_2_ uptake which is greater than the weighted sum of the uptakes for the pure solvent and the porous host in its solid form.^18–21^ In that case the discrepancy was ascribed to there being good binding sites for CO_2_ molecules on the outer surface of the host which are not available for gas binding in the solid form of the host due to crystal packing. We also note that a study of gas uptake kinetics into Type III PLs has pointed to the significance of an adsorbed layer (*i.e.* solvation shell) around MOF particles dispersed in a carrier liquid in determining gas uptake kinetics.^22^ Taking these reference points together with the current work, it suggests that the interface between the solvent and the host in PLs can be important in determining overall behaviour (both kinetic and thermodynamic) with regard to the dissolution of gases. Greater elucidation of this region will be important in more fully understanding and exploiting gas uptake in PLs. Also, intriguingly, it suggests that in developing PLs toward high gas selectivity, there may be mechanisms to reduce, as well as increase, the solubility of a given gas.

## Author contributions

CFK conducted the synthetic and analytical work. Gas solubility measurements were carried out by CFK and EMFR. SFF, JLB, and MDdP conducted the MD simulations. KTH contributed to the ideation. SLJ obtained funding, contributed to ideation and supervised the project. The paper was drafted jointly by CFK, SLJ, MGdP, SFF, and JLB. All authors have given approval to the final version of the manuscript.

## Conflicts of interest

There are no conflicts to declare.

## Supplementary Material

SC-OLF-D5SC06588G-s001

SC-OLF-D5SC06588G-s002

SC-OLF-D5SC06588G-s003

## Data Availability

The supporting data of this manuscript are available in the supplementary information (SI). Supplementary information is available. See DOI: https://doi.org/10.1039/d5sc06588g. Computational modelling data are deposited here: C. F. Kelly, É. M. F. Rooney, S. Fonrouge, J. L. Borioni, M. G. Del Pópolo and S. L. James, DOI: https://doi.org/10.5281/zenodo.14560136.
