# Supplementary material for: Gas exclusion zones in Type II porous liquids
Source: Chem Sci. 2025 Nov 19;17(2):1341–8. doi: 10.1039/d5sc06588g (PMC12649794; doi:10.1039/d5sc06588g)
Supplement: SC-017-D5SC06588G-s001 [file SC-017-D5SC06588G-s001.pdf]

Supplementary Information (SI) for Chemical Science.
This journal is © The Royal Society of Chemistry 2025

Gas Exclusion Zones in Type II Porous Liquids

Cathal F. Kelly,[a] Sergio F. Fonrouge,[b] José L. Borioni,[c] Mario G. Del Pópolo,[b] Émer M. F. Rooney,[a] Deborah E. Crawford,[d] K. Travis Holman,[e] and Stuart L. James*[a]

Supplementary Information

S1 Synthesis of Noria$_{OEt}$

Noria$_{OEt}$ was synthesised according to the method described by Alexander *et al*[1]. To a RBF, 3-ethoxyphenol (11.0 g, 79.6 mmol), TFA (10 mL, 130.7 mmol) and CHCl3 were added and agitated for 10 mins. Glutyraldehyde (1,5-pentanedial, 50% in H_2O) (3.66 mL, 20.2 mmol) was added slowly and the reaction mixture heated to reflux at 80°C for 48 h, cooled to rt and poured into methanol (150 mL). The suspension was filtered through a 4-pore sinter funnel with nylon filter paper and washed several times with Et_2O to afford Noria$_{OEt}$ as a pale cream solid. Which was dried in vacuum at 80 °C overnight (yield 4.39 g, 65%).

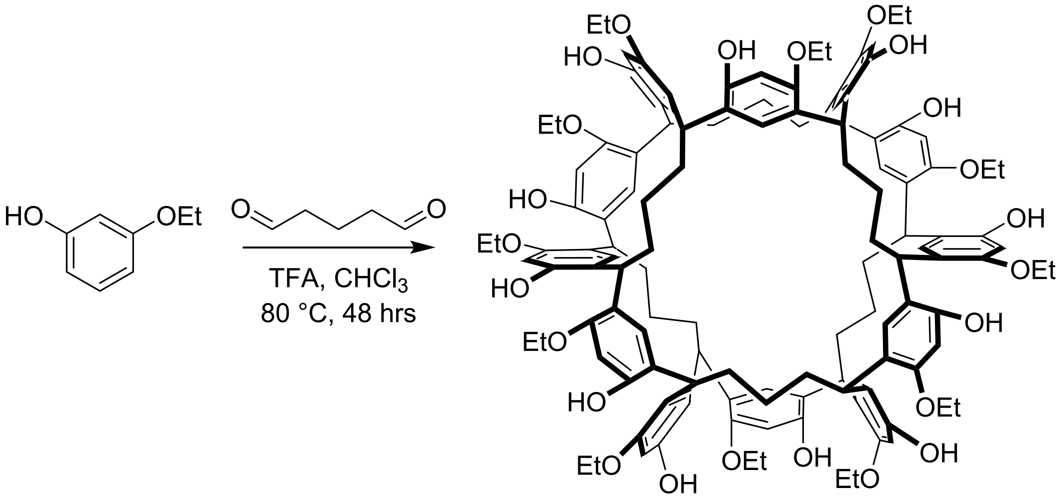

Figure S 1 Synthesis of Noria$_{OEt}$

Analytical data were in agreement with literature.[1]

S2 Synthesis of Cryptophane-A

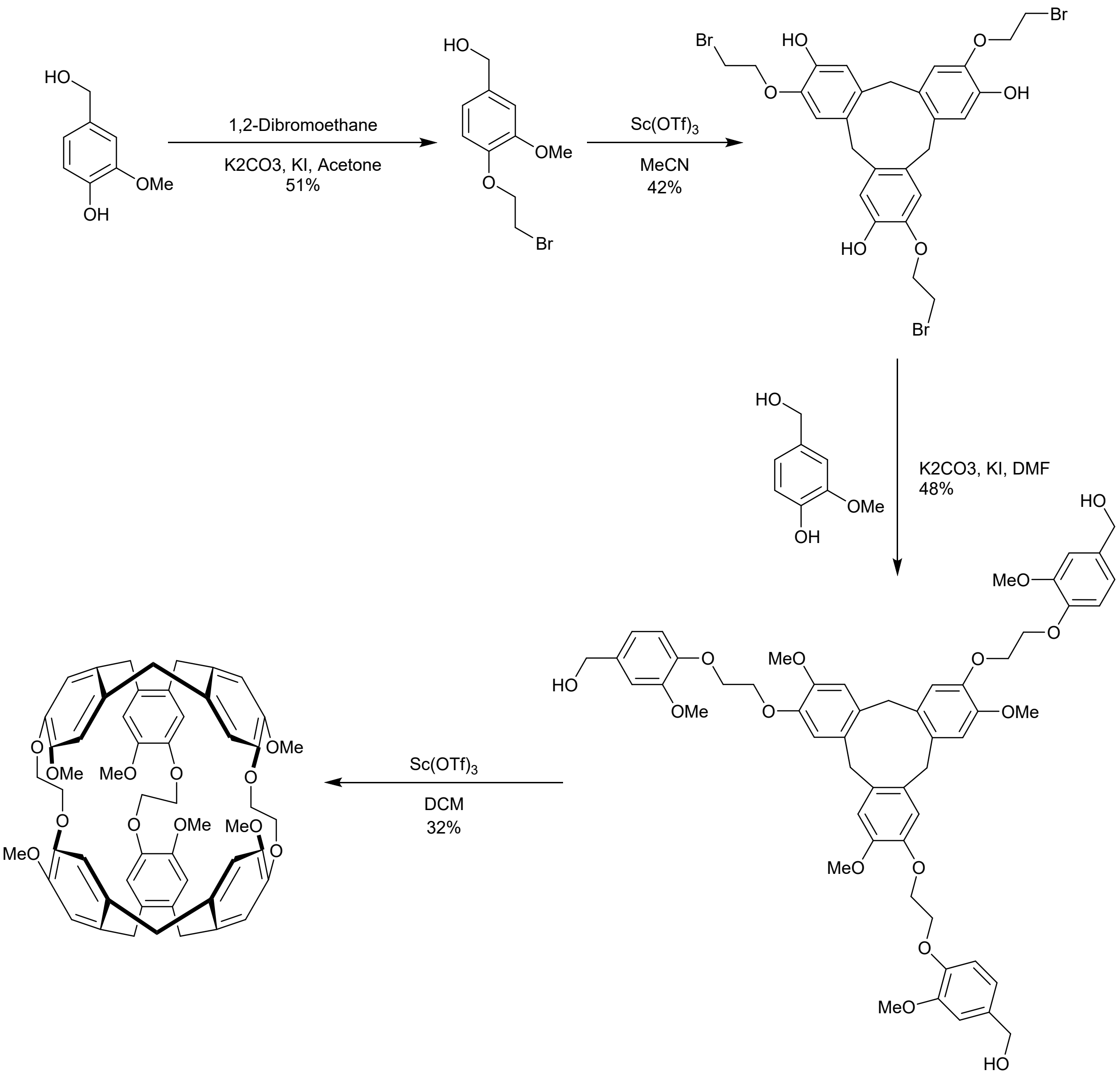

Figure S 2 Synthesis of Cryptophane-A.

Synthesis of (4-(bromoethyl)-3-methoxyphenyl)methanol

Vanillyl alcohol (10 g, 64.87 mmol) was dissolved in acetone (100 mL) in a round bottomed flask. To the flask was added potassium carbonate (44.8 g, 324.14 mmol) and 1,2-dibromoethane (56.3 mL, 24.94 g, 132.76 mmol) and the reaction mixture stirred at reflux overnight. After being allowed to cool to room temperature, the reaction mixture was filtered, and the filtrate was concentrated *in vacuo*. The resulting solid residue was recrystallised from Et_2O with hexane to yield the product as a white solid (8.69 g, 33.28 mmol, 51.3% yield).

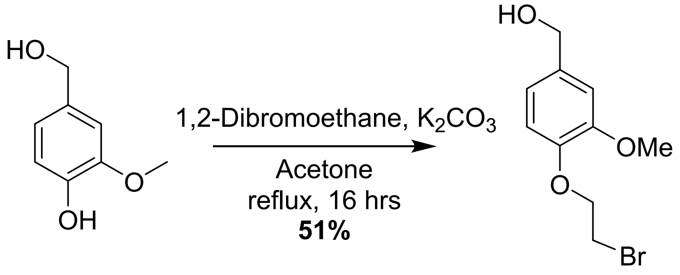

Figure S 3 Synthesis of (4-(bromethyl)-3-methoxyphenyl)methanol.

Product confirmed by comparison with spectrum reported by Wei *et al.*.[2]

Synthesis of tris(*o*-bromoethyl)cyclotriguaiacylene

Scandium (III) triflate (0.27 g, 0.57 mmol) was added to a solution of (4-(bromoethyl)-3-methoxyphenyl)methanol (5 g, 19.15 mmol) in dry acetonitrile (40 mL) under argon. The reaction mixture stirred overnight at 60 °C. The reaction was confirmed as being completed by TLC analysis. The reaction mixture was concentrated *in vacuo* and purified using column chromatography (100% DCM to 5% Et_2O in DCM). The desired product was obtained as a white solid (1.92 g, 2.63 mmol, 41.2% yield).

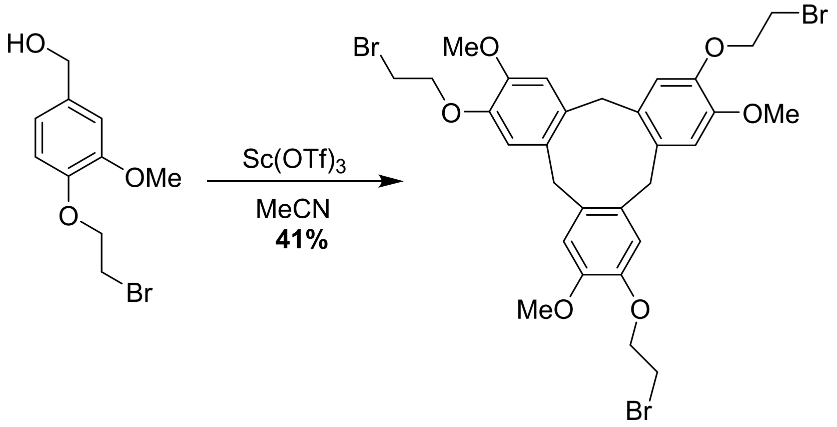

Figure S 4 Synthesis of tris(O-bromoethyl)cyclotriguaiacylene.

Product confirmed by comparison with spectrum reported by Taratula *et al.*.[3]

Synthesis of tris(*o*-ethyloxy-vanillyl alcohol)cyclotriguaiacylene

Vanillyl alcohol (2.49 g, 16.12 mmol), caesium carbonate (6.95 g, 21.33 mmol) and potassium iodide (0.01 g, 0.06 mmol) were added to a solution of tris(O-bromoethyl)cyclotriguaiacylene (3.46 g, 4.74 mmol) in anhydrous DMF (87.5 mL). The resulting solution stirred overnight at 80 °C, after which the mixture was allowed to cool to room temperature. The mixture was concentrated *in vacuo*, distilled water was added (100 mL), and the aqueous mixture was extracted with EtOAc (2 x 100 mL). The combined organic phases were washed with 10% aqueous NaOH (2 x 100 mL), water (2 x 100 mL), and brine (100 mL). The organic phase was dried over Na_2SO_4, filtered and the solvent was removed under vacuum to afford the crude product which was purified by column chromatography to yield the product as an off-white solid (2.16 g, 2.28 mmol, 48.1% yield).

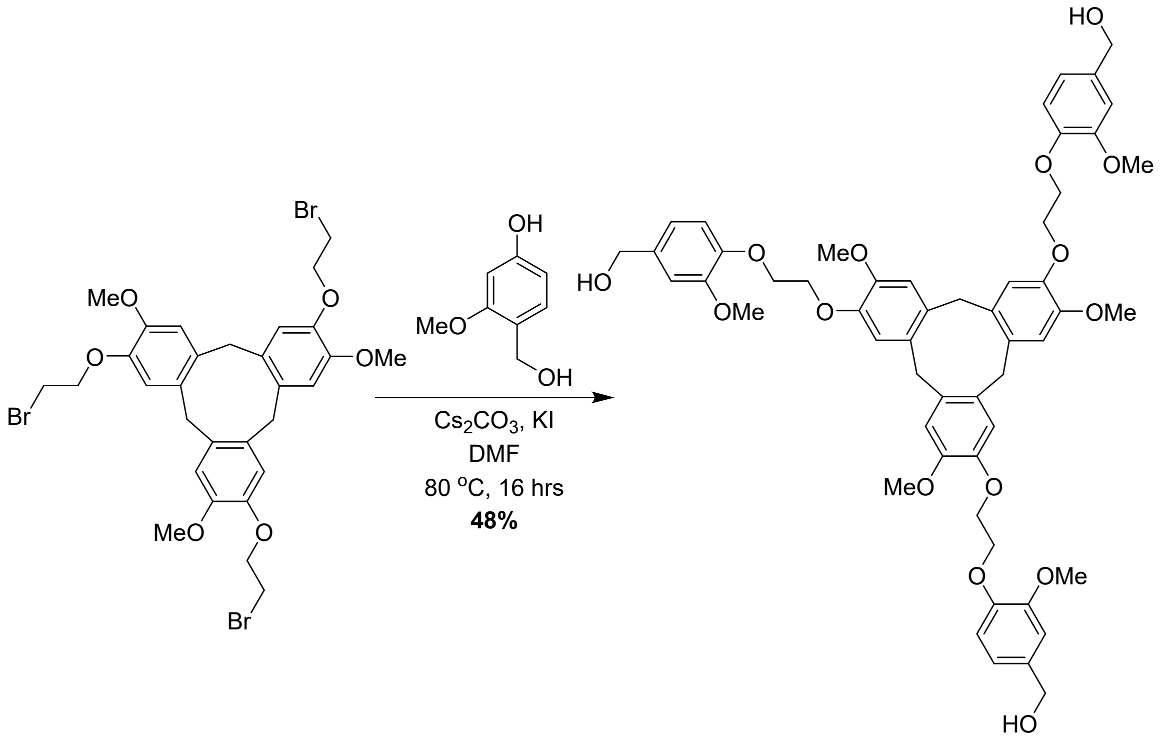

Figure S 5 Synthesis of tris(O-ethyloxy-vanillyl alcohol)cyclotriguaiacylene.

Product confirmed by comparison with spectrum reported by Della-Negra *et al.*.[4]

Synthesis of Cryptophane-A

A solution of tris(O-ethyloxy-vanillyl alcohol)cyclotriguaiacylene (2.16 g, 2.28 mmol) in anhydrous DCM (650 mL) was added at a rate of 60 µL/min to a suspension of scandium (III) triflate (1.12 g, 2.28 mmol) in anhydrous DCM (200 mL) at 60 °C under argon. The resulting suspension continued to stir for a further day before being concentrated *in vacuo* and purified by column chromatography (50% DCM in Hexane to 100% DCM). The desired product was obtained as a white powder (0.65 g, 0.72 mmol, 32% yield).

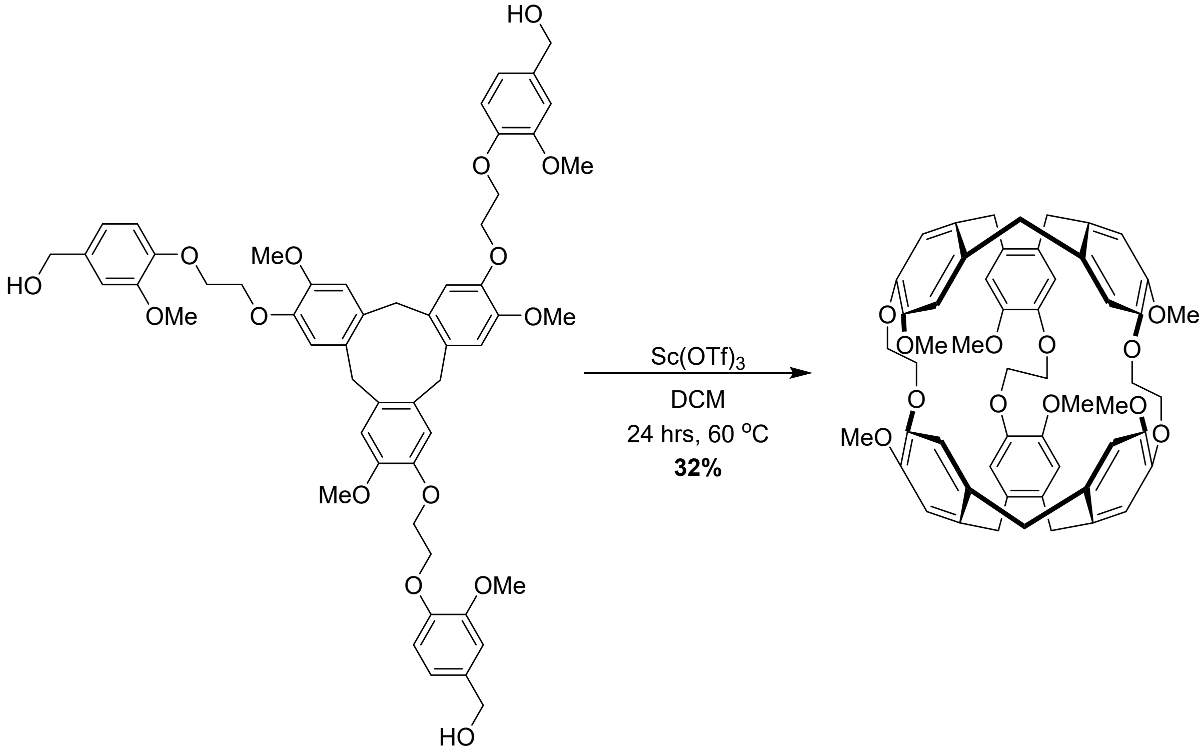

Figure S 6 Synthesis of Cryptophane-A.

Product confirmed by comparison with spectra reported by Canceill *et al.*.[5]

S3 Generation of Type-2 Porous Liquids

Noria$_{OEt}$ was dried in a vacuum oven at 80 °C overnight and 15-crown-5 was dried by stirring over calcium hydride before being filtered and stored under argon. Noria$_{OEt}$ (1.60 g, 0.48 mmol) was dissolved in 15-crown-5 (20 mL) using gentle heating. The resulting solution was monitored overnight to check that no precipitation occurred.

Cryptophane-A was dried in a vacuum oven at 80 °C overnight and Cyrene was distilled over $MgSO_4$ and stored under argon. Cryptophane-A (0.86 g, 0.96 mmol) was dissolved in Cyrene (20 mL) using gentle heating. The resulting solution was left overnight to monitor any potential precipitation.

S4 Barometric Gas Solubility Measurements

The solubility measurements of CH_4 and CO_2 in Cyrene and a solution of cryptophane-A in Cyrene at 298.15 K from 1-5 bar, as well as CO_2 in 15C5 and in a solution of $Noria_{OEt}$ in 15C5 at 298.15 K from 1-5 bar were measured using the barometric gas rig apparatus shown in Figure S7. The general method has been described elsewhere.[1] The process makes use of a constant temperature and pressure while directly measuring the volume of gas needed to maintain the pre-set conditions. The volume of gas needed directly corresponds to the amount gas adsorbed by the materials. The gas solubility measurements were allowed to rest at each pressure point for 1 hour to ensure equilibration. Samples were added into the sample cell and degassed at room temperature for an hour by vacuum.

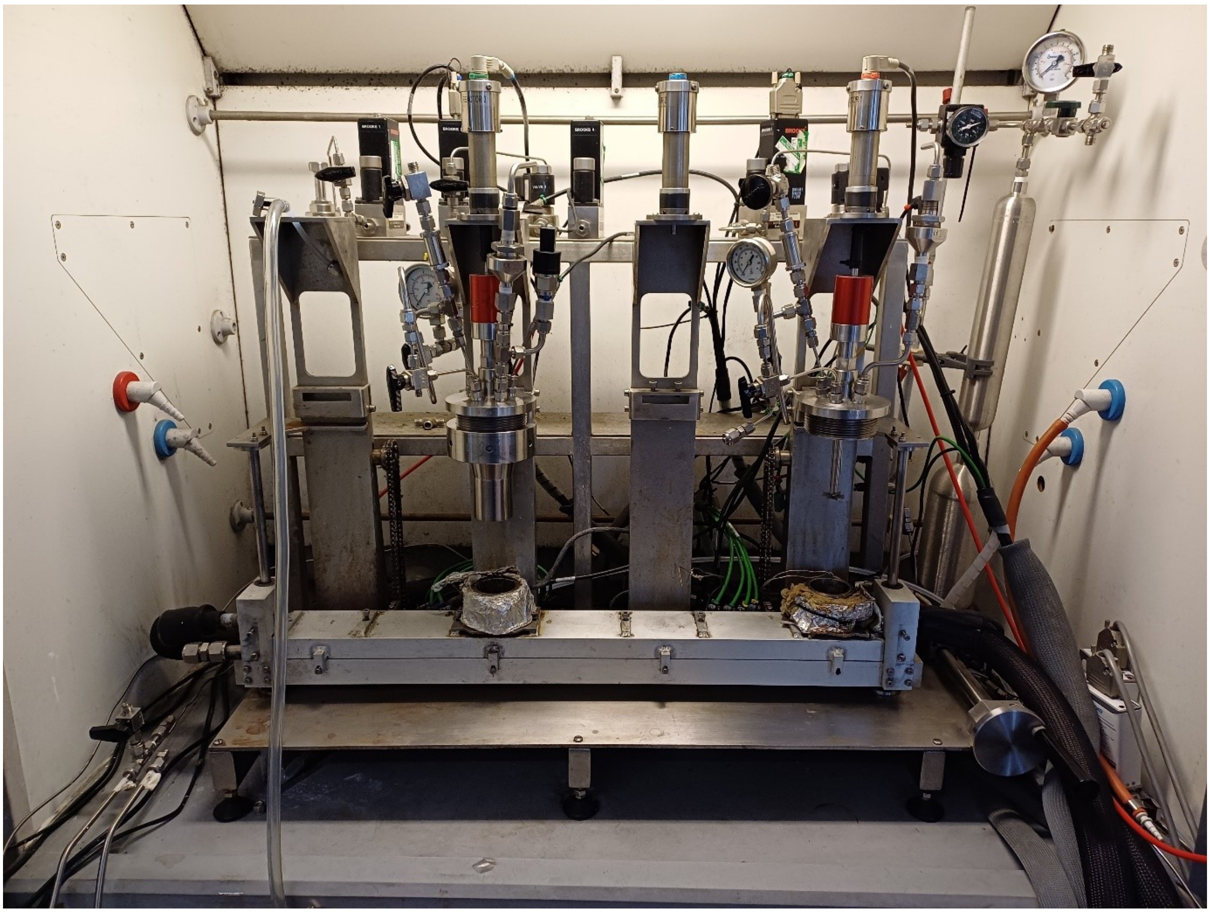

Figure S7 Barometric gas rig apparatus used to measure gas solubilities.

Table S1 CH_4 uptake of 15C5 at 303.15 K from 1-5 bar (to 4 decimal places). Reproduced from Alexander et al.[1]

Pressure (bar)	1st Run (mg/g)	2nd Run (mg/g)	3rd Run (mg/g)	Average CO_2 Uptake of 15C5 (mg/g)	Standard Deviation
1	0.1322	0.1125	0.1338	0.1262	0.0097
2	0.2625	0.236	0.2527	0.2504	0.0109
3	0.4177	0.373	0.464	0.4182	0.0372
4	0.5944	0.5434	0.7126	0.6168	0.0709
5	0.852	0.8194	0.9836	0.8850	0.0710

Table S2 CH_4 uptake of $Noria_{OEt}$ in 15C5 (0.024 M) at 303.15 K from 1-5 bar (to 4 decimal places). Reproduced from Alexander et al.[1]

Pressure (bar)	1st Run (mg/g)	2nd Run (mg/g)	3rd Run (mg/g)	Average CH_4 Uptake of $Noria_{OEt}$ in 15C5 (mg/g)	Standard Deviation
1	0.2685	0.2276	0.1443	0.2135	0.0517
2	0.4708	0.3755	0.3515	0.3993	0.0515
3	0.7056	0.6568	0.5704	0.6443	0.0559
4	0.8553	0.8551	0.8313	0.8472	0.0113
5	1.1552	1.2721	1.1322	1.1865	0.0613

Table S3 CO_2 uptake of 15C5 at 298.15 K from 1-5 bar (to 4 decimal places).

Pressure (bar)	1st Run (mg/g)	2nd Run (mg/g)	3rd Run (mg/g)	Average CO_2 Uptake of 15C5 (mg/g)	Standard Deviation
1	4.2271	3.9762	3.8242	4.0092	0.1661
2	8.5589	7.9063	8.1459	8.2037	0.2695
3	12.2655	12.0047	12.1387	12.136	0.1065
4	16.2261	16.2122	15.8179	16.085	0.1892
5	20.3564	21.1353	19.8335	20.4417	0.5348

Table S4 CO_2 uptake of $Noria_{OEt}$ in 15C5 (0.024 M) at 298.15 K from 1-5 bar (to 4 decimal places).

Pressure (bar)	1st Run (mg/g)	2nd Run (mg/g)	3rd Run (mg/g)	Average CH_4 Uptake of $Noria_{OEt}$ in 15C5 (mg/g)	Standard Deviation
1	3.4418	2.3991	3.4435	3.0948	0.4920
2	6.5832	5.4207	6.4306	6.1448	0.5158
3	9.3750	8.9306	9.7724	9.3593	0.3439
4	12.6422	12.4386	13.3175	12.7994	0.3756
5	16.3057	15.8551	16.3860	16.1823	0.2337

Table S5 CH_4 uptake of Cyrene at 298.15 K from 1-5 bar (values given to 4 decimal places)

Pressure (bar)	1st Run (mg/g)	2nd Run (mg/g)	3rd Run	Average CH_4 Uptake	Standard Deviation

			(mg/g)	of Cyrene (mg/g)	
**1**	0.1401	0.1370	0.1022	0.1265	0.0172
**2**	0.3251	0.3200	0.267	0.3040	0.0263
**3**	0.4916	0.5068	0.3808	0.4597	0.0562
**4**	0.8173	0.7593	0.5919	0.7228	0.0956
**5**	1.0808	1.0335	0.9546	1.0229	0.0521

Table S6 CO_2 uptake of Cyrene at 298.15 K from 1-5 bar (values given to 4 decimal places)

**Pressure (bar)**	**1st Run (mg/g)**	**2nd Run (mg/g)**	**3rd Run (mg/g)**	**Average CH_4 Uptake of $Noria_{OEtOPr}$ in Cyrene (mg/g)**	**Standard Deviation**
**1**	5.8139	6.2504	6.1345	6.1925	0.0579
**2**	11.2097	11.6113	11.7248	11.6681	0.0568
**3**	16.5819	16.3878	17.0036	16.6957	0.3079
**4**	21.9979	22.2826	22.6512	22.4669	0.1843
**5**	27.7262	27.9813	28.1032	28.0422	0.0610

Table S7 CH_4 uptake of Cryptophane-A in Cyrene (0.048 M) at 298.15 K from 1-5 bar (values given to 4 decimal places)

**Pressure (bar)**	**1st Run (mg/g)**	**2nd Run (mg/g)**	**3rd Run (mg/g)**	**Average CH_4 Uptake of Cryptophane-A in Cyrene (mg/g)**	**Standard Deviation**
**1**	0.7177	0.8807	0.5876	0.7992	0.0814
**2**	1.0016	1.2930	0.8398	1.1473	0.1457
**3**	1.3770	1.6122	1.2781	1.4946	0.1176
**4**	1.7526	1.8941	1.6813	1.8233	0.0707
**5**	2.1524	2.2748	2.074	2.2136	0.0612

S8 CO_2 uptake of Cryptophane-A in Cyrene (0.048 M) at 298.15 K from 1-5 bar (values given to 4 decimal places)

**Pressure (bar)**	**1st Run (mg/g)**	**2nd Run (mg/g)**	**3rd Run (mg/g)**	**Average CO_2 Uptake of 15C5 (mg/g)**	**Standard Deviation**
**1**	7.7252	7.5307	7.1697	7.4752	0.2301
**2**	14.5045	13.9041	13.2513	13.8866	0.5117
**3**	20.6504	19.3953	18.6633	19.5697	0.8205
**4**	26.4459	24.3006	23.6330	24.7932	1.2000
**5**	31.8303	29.3717	28.7600	29.9874	1.3268

S5 Capillary NMR Experiments

1.5 mL of either Cyrene or Cryptophane-A in Cyrene (0.048 M) was added to an NMR tube along with a sealed capillary of $(CD_3)_2CO$. A host gas (CH_4 or C_2H_6) was then bubbled through the sample for 1 hour. The sample was sealed with parafilm and an NMR spectrum obtained. The resulting spectra can be seen below, with the peak corresponding to the studied gas highlighted.

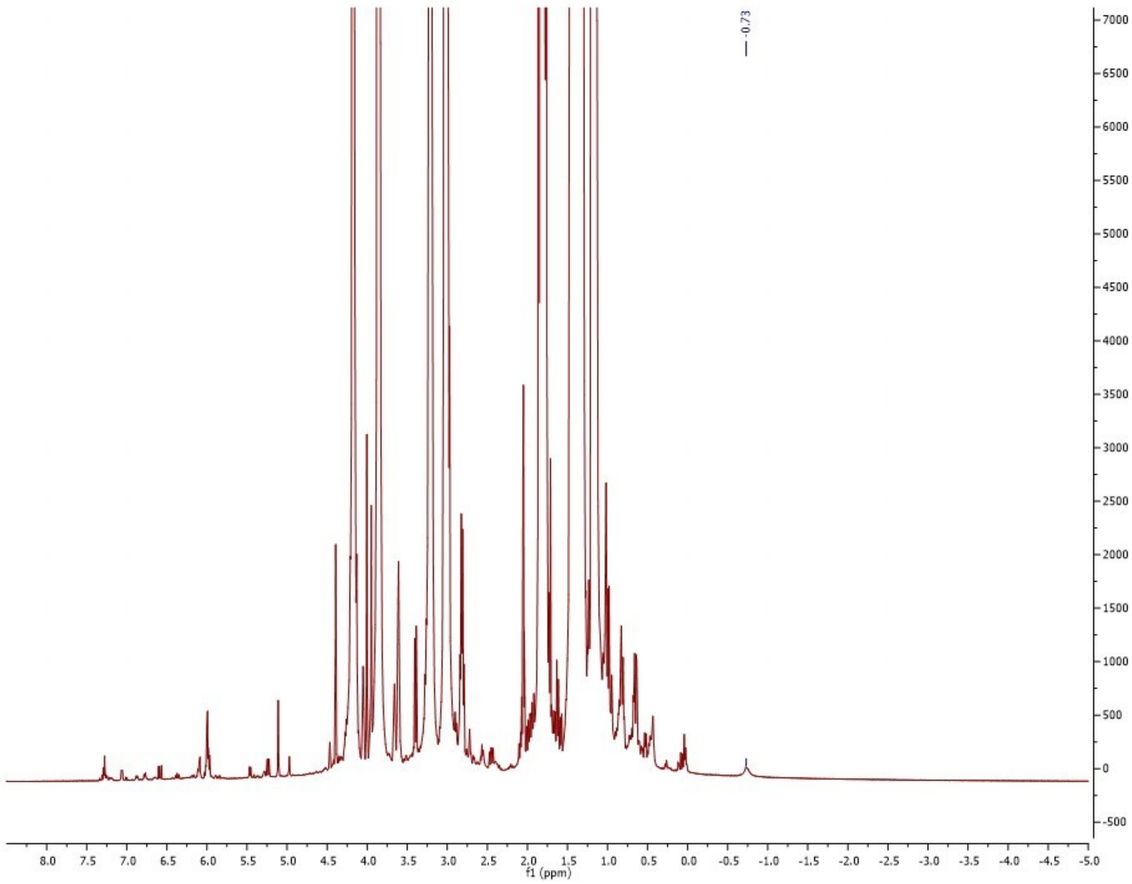

Figure S 8 ^{1}H-NMR spectrum of CH_4 bubbled through Cyrene using a deuterated acetone capillary (400 MHz, $(CD_3)_2CO$))

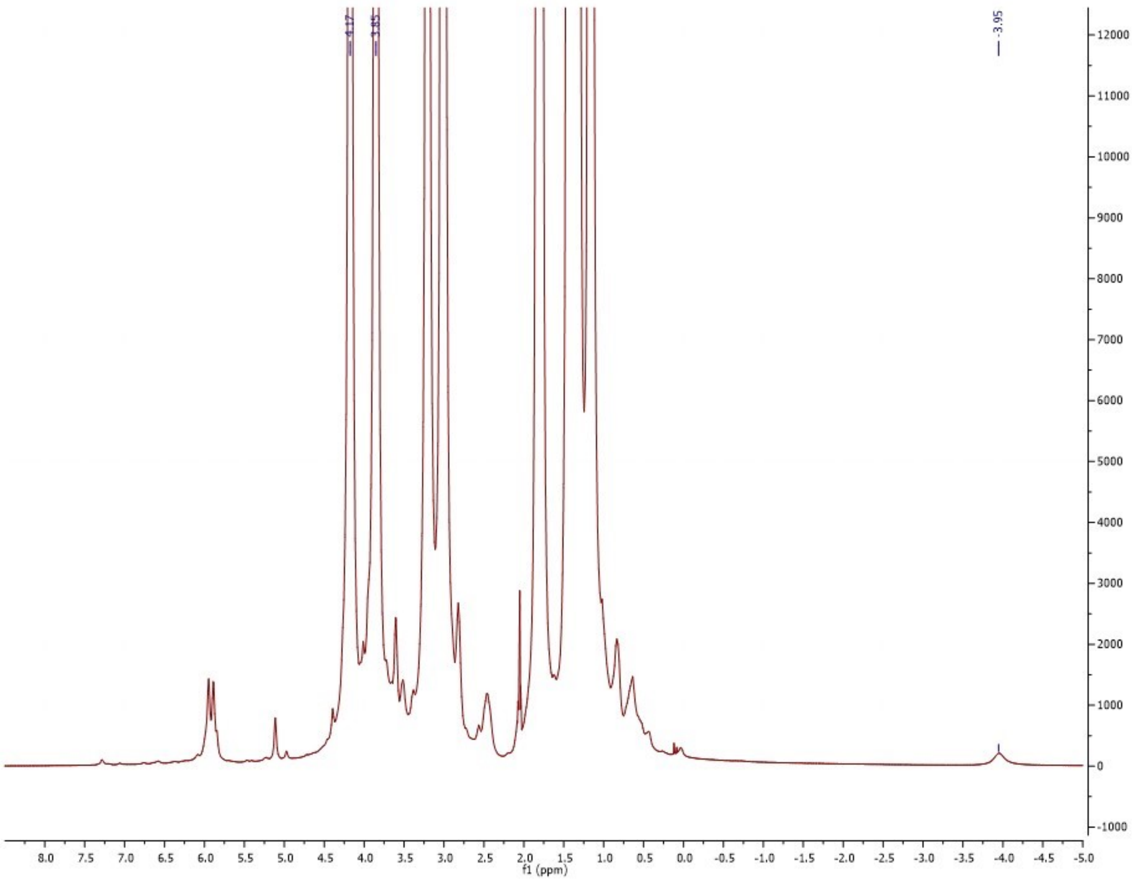

Figure S 9 ^{1}H-NMR spectrum of CH_4 bubbled through Cryptophane-A in Cyrene (0.048 M) using a deuterated acetone capillary (400 MHz, $(CD_3)_2CO$))

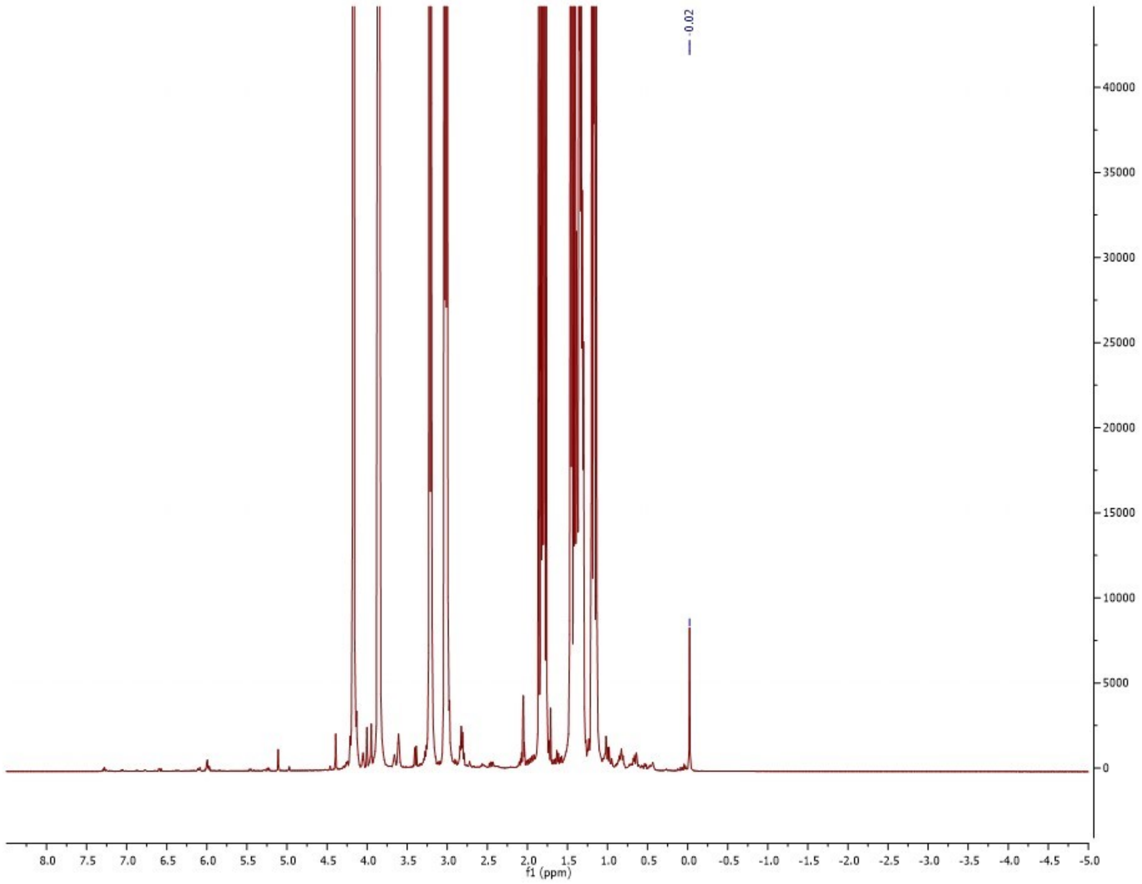

Figure S 10 ^{1}H-NMR spectrum of C_2H_6 bubbled through Cyrene using a deuterated acetone capillary (400 MHz, $(CD_3)_2CO$))

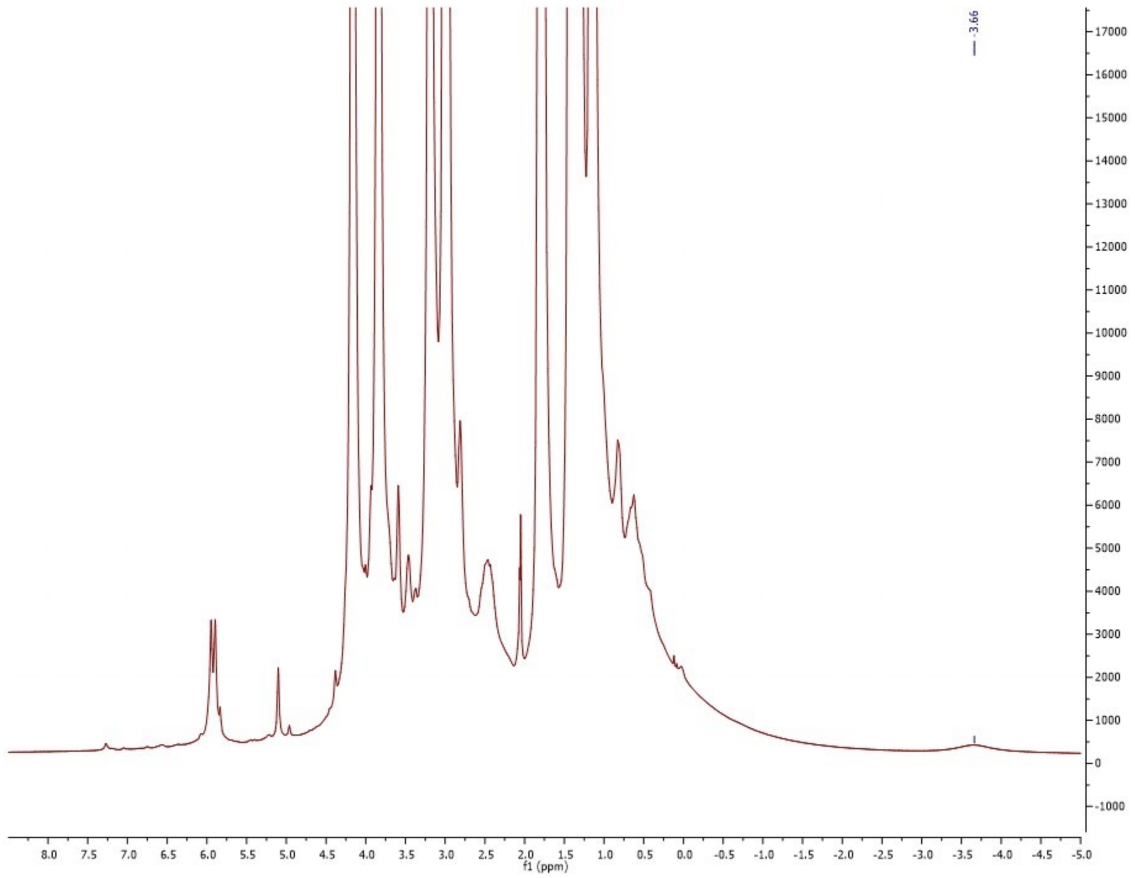

Figure S 11 ^{1}H-NMR spectrum of C_2H_6 bubbled through Cryptophane-A in Cyrene (0.048 M) using a deuterated acetone capillary (400 MHz, $(CD_3)_2CO$))

S6 MD Simulations

MD simulations were conducted for systems that contained a single $Noria_{OEt}$ cage, 1000 15-crown-5 ether molecules, and a fixed amount of gas molecules matching experimental gas concentrations (Table S 1-4), or no gas molecules at all. These systems were simulated with *Gromacs-2022.3* in the isothermic, isobaric ensemble (NPT) at 300 K and 1 or 5 bar using the Bussi-Donadio-Parrinello (v-rescale) thermostat and the Parrinello-Rahman barostat, for which the time constants tau-t and tau-p were set to 0.5 and 1.0 ps respectively[6]. Integration was done with a leap-frog algorithm (md) and a timestep of 1 fs. A general overview of these simulations is provided in Table S5, and the force-field parameters for the gases are detailed in section S7. In addition, input files are available in the Zenodo online repository[7]. Trajectories were visualised with *VMD v1.9*[8].

Table S5 Loading conditions of unbiased MD simulations. These comprised at least a single $Noria_{OEt}$ cage in 1000 solvent molecules and were run in the NPT ensemble at 300 K

**Solute**	**Number of molecules**	**Pressure [bar]**	**Simulation time [ns]**	
			**Equilibration**	**Production**
**None**	n/a	1 and 5	100	200
**CO_2**	23	1	200	1000
	98	5	100	200
**CH_4**	3	1	200	1000
	17	5	100	200

Free energy profiles were obtained from biased MD simulations using the Umbrella Sampling technique (US)[9]. These systems comprised a single $Noria_{OEt}$ cage, 1000 15-crown-5 ether molecules and a single CO_2 or CH_4 molecule. Simulations were run in the NPT ensemble using the same software. The reaction coordinate was defined as the distance between the centres of mass of $Noria_{OEt}$ and the gas molecule. The US sampling was done every 0.1 nm along this reaction coordinate using a harmonic potential and a force constant of 1000 $kJ.mol^{-1}.nm^{-2}$.

It is important to note that the computed free-energy landscape is one-dimensional and based on a spherical reaction coordinate. This approach inherently averages out the effects of external protrusions and indentations in the $Noria_{OEt}$ molecule. A fully three-dimensional free-energy map, though technically and computationally demanding, would likely reveal a more intricate energy landscape, with higher barriers or regions where gas exclusion is even more pronounced, and localized free-energy minima.

S7 Models and Force-field Parameters

The force-field parameters for $Noria_{OEt}$, 15-crown-5, and methane were taken from Alexander *et al.*, where the RESP method was applied to obtain partial atomic charges from the optimized molecular structures[1]. For molecular dynamics (MD) simulations, the parameters for each molecule were taken from the OPLS-AA force-field[10]. Additionally, non-bonded interactions between methane atoms and key $Noria_{OEt}$ atoms were adjusted to correctly reproduce the gas-host binding energies obtained from DFT calculations.

For carbon dioxide, the parameters were taken directly from Jamali *et al.* without any modifications[11].

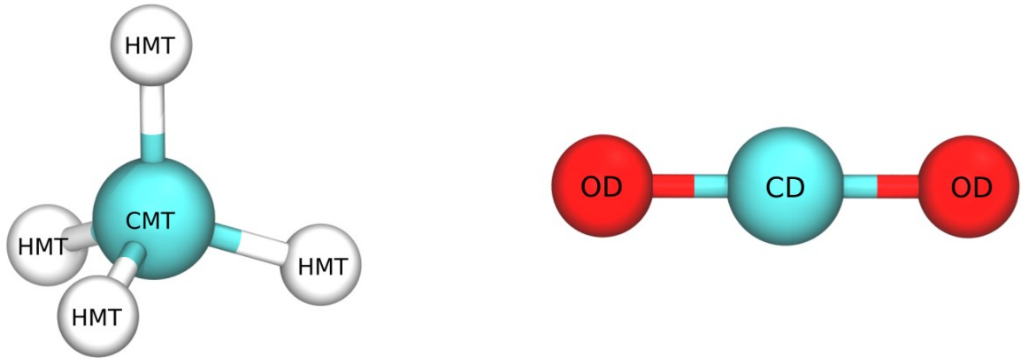

Figure S12 Representation of methane (left) and carbon dioxide (right). The labels correspond to force-field atom types, as defined in Table S6 and S9.

Table S6 Atom types, point charges and Lennard-Jones parameters for methane. Atom type.

Atom type	Mass	Charge (e)	Sigma (nm)	Epsilon (kJ/mol)
**CMT**	12.01100	-0.576308	0.3500000	0.2761440
**HMT**	1.00800	0.144077	0.2500000	0.1255200

Table S7 Bond-stretching parameters for methane.

Bond type	b_o(nm)	K_0 (kJ mol^{-1}nm^{-2})
**CMT-HMT**	0.10900	284512.0

Table S8 Angle parameters for methane.

Angle type	θ_0 (deg)	K_θ (kJ mol^{-1}rad^{-2})
**HMT-CMT-HMT**	107.800	276.144

Table S9 Atom types, point charges and Lennard-Jones parameters for carbon dioxide. Atom type.

Atom type	Mass	Charge (e)	Sigma (nm)	Epsilon (kJ/mol)
**CD**	12.01100	0.6512	0.2800	0.2340520
**OD**	15.99940	-0.3256	0.3028	0.6683170

Table S10 Bond-stretching parameters for carbon dioxide.

Bond type	b_o(nm)	K_0 (kJ $mol^{-1}nm^{-2}$)
**CD-OD**	0.1162	844334.7

Table S11 Bond-stretching parameters for carbon dioxide.

Angle type	θ_0 (deg)	K_0 (kJ $mol^{-1}rad^{-2}$)
**OD-CD-OD**	180.000	903.78

S8 Gas-Host Binding Energies in Vacuum

To evaluate the Lennard-Jones parameters for CO_2-Noria$_{OEt}$ interactions, we computed the energetics of guest-host complex formation in vacuum through Density Functional Theory (DFT) calculations and Molecular Mechanics (MM) simulations. Non-bonded interaction energies between CO_2 and key Noria$_{OEt}$ atoms were computed to characterize four distinct binding sites (Figure S13Table S12). DFT calculations were performed using Gaussian 16 Rev. C.01 and the hybrid-GGA ω-B97XD functional[12,13]. Geometries were optimized in vacuum using the 6-31G(d) basis set, and stationary points were confirmed via Hessian matrix calculations, ensuring all eigenvalues were positive for a minimum. Electronic energies were further refined through single-point calculations at the 6-31+G(d) level.

To assess the accuracy of the force-field parameters for CO_2- Noria$_{OEt}$ interactions, MM formation energies were computed (Table S12). All DFT-optimized geometries were subsequently optimized using the conjugate gradient algorithm in Gromacs in order to assess the corresponding formation energy in the classical force-field. A similar procedure was used for to CH_4- Noria$_{OEt}$ interactions as reported by Alexander *et al*[1]. However, in that case, specific pair-wise interactions were adjusted to fully reproduce the dominant CH_4 binding site at the centre of the Noria$_{OEt}$.

Table S12 Electronic formation energies from DFT and Molecular Mechanics (MM) calculations performed with the Gaussian 16 and Gromacs programs, respectively. Energies correspond to geometry-optimized isolated molecules (vacuum calculations) at 0 K. Binding sites variants are illustrated in Figure S13.

Binding site	DFT (kcal/mol)	MM (kcal/mol)
**1 (inclusion complex)**	-7.41	-7.25
**2**	-7.66	-6.04
**3**	-8.11	-7.23
**4**	-4.74	-3.95

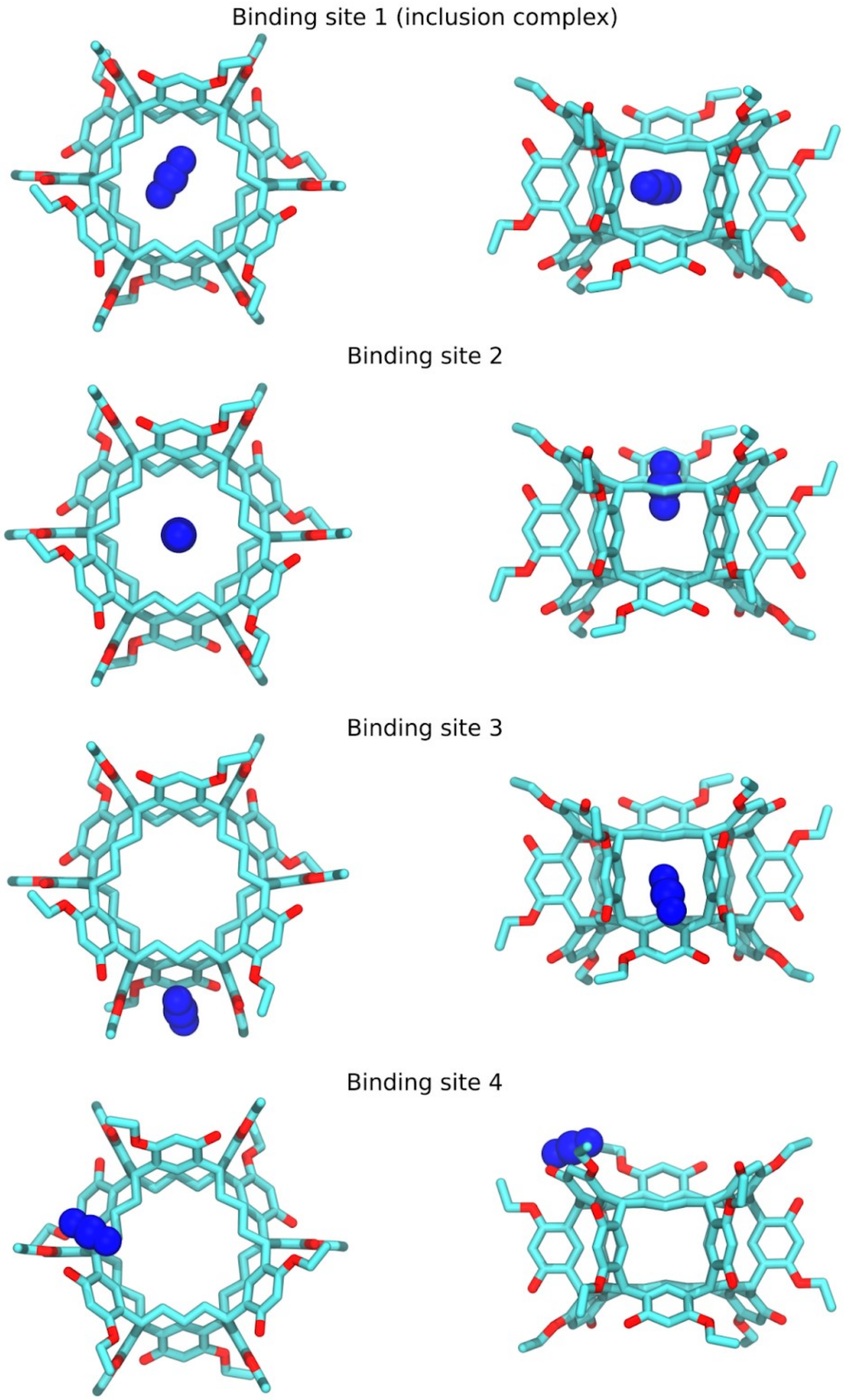

Figure S13 Binding sites explored for CO_2 in Noria$_{OEt}$. The corresponding formation energies are listed in Table S12.

S9 Noria$_{OEt}$ Occupancy

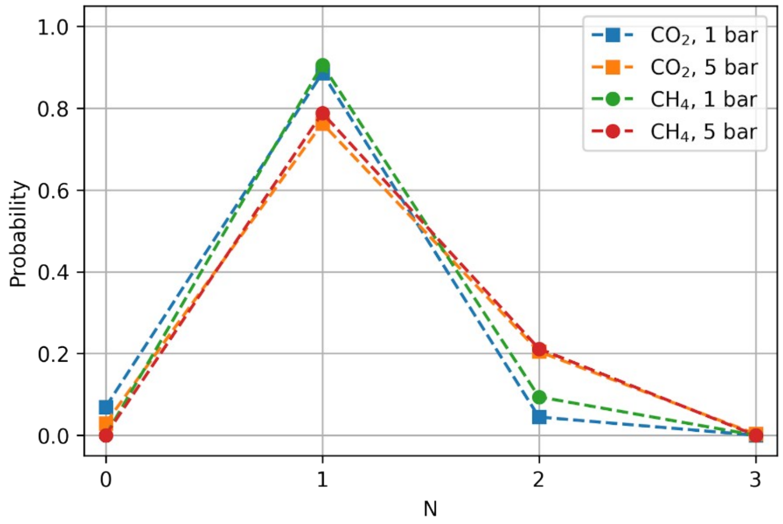

Figure S14 Probability of finding N gas molecules inside the Noria$_{OEt}$ cage throughout the MD simulations listed in Table S5. A gas molecule was deemed to be inside the cage if the distance between their centres of mass was shorter than 0.6 nm. Sampling of occupancy was done on the output trajectories, written in timesteps of 10 ps.

S10 Radial Distribution Functions

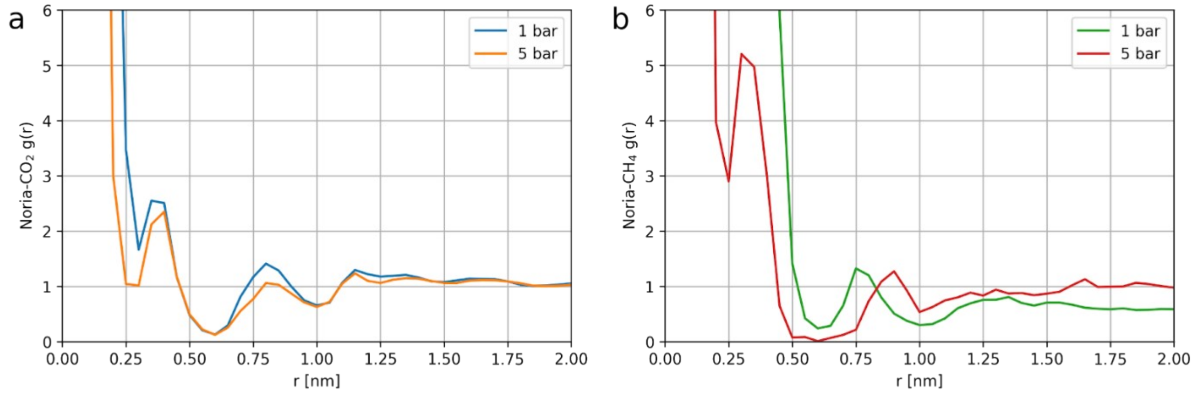

Figure S15 Radial distribution function of CO_2 relative to Noria$_{OEt}$, obtained from MD simulations. The system contained a single Noria$_{OEt}$ host, 1000 15-crown-5 ether molecules, and CO_2 at concentrations matching experimental values. Additional details are provided in Table S5. g(r) < 1 indicates a local gas concentration lower than that in the bulk.

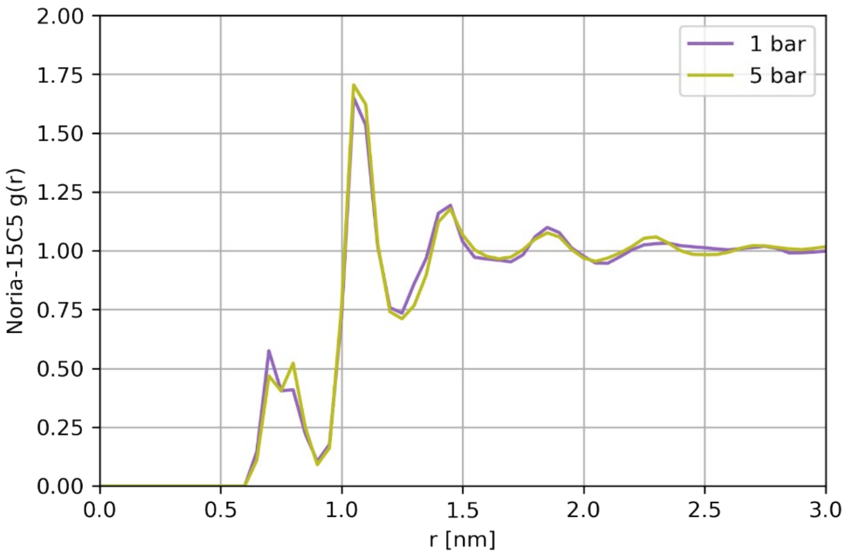

Figure S16 Radial distribution functions (RDFs) between the centre of mass of Noria$_{OEt}$ and the centre of mass of 15C5 were computed at 1 bar and 5 bar from NPT simulations containing a single Noria$_{OEt}$ host in 1000 15C5 molecules, in the absence of CO_2 or CH_4 (simulations details in Table S5). These RDFs characterize the arrangement of solvent molecules around the host, revealing a prominent peak at ~1.1 nm, which is fully enclosed within the identified exclusion zones.

S11 Movies

Movie-1: Time-accumulated positions of the carbon atoms in CO_2 molecules over 200 ns of MD simulation at 5 bar. Red spheres represent CO_2 positions within the exclusion zone, while blue spheres depict their successive locations inside the host cavity. The grey and blue translucent spheres mark the boundaries of the exclusion zone, consistent with Figure 5 of the manuscript. This visualization provides insight into the 3D density distribution of CO_2 molecules in and around Noria$_{OEt}$.

Movie-2: MD simulation movie of the porous liquid loaded with CO_2 at 5 bar. The translucent spheres mark the boundaries of the exclusion zone, consistent with Figure 5 of the manuscript. On the left, a lateral view of Noria$_{OEt}$ is shown, while on the right, an upper view is displayed. CO_2 molecules inside the inner shell appear in blue, those in the outer shell in red, and those in the solvent region in green. Solvent molecules are hidden for clarity.

S12 Distribution of Cavities around the Host

To quantify the spatial distribution of cavities around Noria$_{OEt}$, we computed the radial distribution function of large cavities, $g_{cav}(r)$, relative to the geometric centre of the host molecule. This analysis was based on 100 configurations extracted from MD simulations of the porous liquid at 300 K and 1 bar. Cavities were identified using a hard-sphere insertion approach, where a grid was constructed along the x, y, and z axes of the simulation cell with grid points spaced 0.1 nm apart. At each grid point, we determined the largest sphere with radius R > 0.1 nm that could be inserted without overlapping the van der Waals radii of any surrounding atoms. Once the cavity positions were identified, their distances from the geometric centre of Noria$_{OEt}$ were computed to construct $g_{cav}(r)$. This function was normalized using the average density of cavities with R > 0.1 nm for each individual MD snapshot.

S13 Estimation of van der Waals and cavity volumes via Monte Carlo sampling

To estimate the van der Waals (vdW) volume of each molecule, we employed a Monte Carlo integration scheme. Random points were uniformly sampled within a bounding box that enclosed the entire molecule, extended by 4 Å in each spatial direction to avoid truncating peripheral volume. For each

point, we checked whether it fell within the vdW radius of any atom in the molecule. The vdW volume was then calculated as the fraction of points falling within any vdW sphere, multiplied by the volume of the bounding box. It is important to note that this estimate includes only the space directly occupied by the atoms and does not include the internal cavity of the molecule.

To compute the cavity volume, we first identified the atoms forming the rigid inner cage of the molecule and constructed the convex hull defined by their coordinates. We then used the same random point set to identify those points that were located inside the convex hull but outside the vdW spheres of all atoms. The volume of the cavity was estimated as the fraction of such points multiplied by the bounding box volume. This approach yields the internal volume that is geometrically enclosed by the cage but not occupied by any atom. For Noria, the vdW volume was found to be 1792.56 Å^3, with a cavity volume of 140.69 Å^3. For Cryptophane, the vdW volume was 764.86 Å^3, and the cavity volume was 123.79 Å^3. By construction, the vdW and cavity volumes are non-overlapping, and their sum provides a lower-bound estimate of the total molecular envelope. The Noria$_{OEt}$ volumes were calculated using the constructed model. Cryptophane-A models were obtained using crystallographic data reported by Taratula *et al*[14].

References

1 F. M. Alexander, S. F. Fonrouge, J. L. Borioni, M. G. Del Pópolo, P. N. Horton, S. J. Coles, B. P. Hutchings, D. E. Crawford and S. L. James, *Chem. Sci.*, 2021, **12**, 14230–14240.

2 Q. Wei, G. K. Seward, P. A. Hill, B. Patton, I. E. Dimitrov, N. N. Kuzma and I. J. Dmochowski, *J. Am. Chem. Soc.*, 2006, **128**, 13274–13283.

3 O. Taratula, P. A. Hill, Y. Bai, N. S. Khan and I. J. Dmochowski, *Org. Lett.*, 2011, **13**, 1414–1417.

4 O. Della-Negra, Y. Cirillo, T. Brotin, J.-P. Dutasta, P.-L. Saaidi, B. Chatelet and A. Martinez, *Chem. Commun.*, 2022, **58**, 3330–3333.

5 J. Canceill, A. Collet, G. Gottarelli and P. Palmieri, *J. Am. Chem. Soc.*, 1987, **109**, 6454–6464.

6 M. J. Abraham, T. Murtola, R. Schulz, S. Páll, J. C. Smith, B. Hess and E. Lindahl, *SoftwareX*, 2015, **1–2**, 19–25.

7 C. F. Kelly, É. M. F. Rooney, S. Fonrouge, J. L. Borioni, M. G. Del Pópolo and S. James, DOI:10.5281/zenodo.14560136.

8 W. Humphrey, A. Dalke and K. Schulten, *J. Mol. Graph.*, 1996, **14**, 33–38.

9 D. Frenkel and B. Smit, *Understanding Molecular Simulation*, Academic Press Inc., 2nd edn., 2001.

10 W. L. Jorgensen, D. S. Maxwell and J. Tirado-Rives, *J. Am. Chem. Soc.*, 1996, **118**, 11225–11236.

11 S. H. Jamali, M. Ramdin, T. M. Becker, S. K. Rinwa, W. Buijs and T. J. H. Vlugt, *J. Phys. Chem. B*, 2017, **121**, 8367–8376.

12 Frisch, Gaussian 16, Revision C.01.

13 J.-D. Chai and M. Head-Gordon, *Phys. Chem. Chem. Phys.*, 2008, **10**, 6615–6620.

14 O. Taratula, P. A. Hill, N. S. Khan, P. J. Carroll and I. J. Dmochowski, *Nat. Commun.*, 2010, **1**, 148.
